# The Pmt2p-Mediated Protein *O*-Mannosylation Is Required for Morphogenesis, Adhesive Properties, Cell Wall Integrity and Full Virulence of *Magnaporthe oryzae*

**DOI:** 10.3389/fmicb.2016.00630

**Published:** 2016-05-02

**Authors:** Min Guo, Leyong Tan, Xiang Nie, Xiaolei Zhu, Yuemin Pan, Zhimou Gao

**Affiliations:** Department of Plant Pathology, College of Plant Protection, Anhui Agricultural UniversityHefei, China

**Keywords:** *Magnaporthe oryzae*, *O*-mannosylation, conidia germination, appressoria formation, cell wall integrity, pathogenicity

## Abstract

Protein *O*-mannosylation is a type of *O*-glycosylation that is characterized by the addition of mannose residues to target proteins, and is initially catalyzed by evolutionarily conserved protein *O*-mannosyltransferases (PMTs). In this study, three members of PMT were identified in *Magnaporthe oryzae*, and the pathogenic roles of MoPmt2, a member of PMT2 subfamily, were analyzed. We found that MoPmt2 is a homolog of *Saccharomyces cerevisiae* Pmt2 and could complement yeast Pmt2 function in resistance to CFW. Quantitative RT–PCR revealed that *MoPmt2* is highly expressed during conidiation, and targeted disruption of *MoPmt2* resulted in defects in conidiation and conidia morphology. The *MoPmt2* mutants also showed a distinct reduction in fungal growth, which was associated with severe alterations in hyphal polarity. In addition, we found that the *MoPmt2* mutants severely reduced virulence on both rice plants and barley leaves. The subsequent examination revealed that the fungal adhesion, conidial germination, CWI and invasive hyphae growth in host cells are responsible for defects on appressorium mediated penetration, and thus attenuated the pathogenicity of *MoPmt2* mutants. Taken together, our results suggest that protein *O*-mannosyltransferase MoPmt2 plays essential roles in fungal growth and development, and is required for the full pathogenicity of *M. oryzae*.

## Introduction

The filamentous fungus *Magnaporthe oryzae*, which causes rice blast disease, is the most destructive pathogen of cultivated rice plants worldwide (Howard and Valent, 1996). It has been developed as a model system to study fungus–plant interactions due to its economic and scientific importance (Talbot, 2003; Xu et al., 2007; Dean et al., 2012). During growing seasons, like many other phytopathogens, asexual spores play an important role in the disease cycle of *M. oryzae* (Lee et al., 2006). When landed on the plant surface, asexual spores immediately secrets conidial tip mucilage to adhere themselves on rice leaves. Under suitable condition, conidia begin to germinate, and four to 6 h later, a dome-shaped infection structure known as appressorium differentiates at the tip of the germ tube. Rice blast fungus generates enormous amount of turgor pressure (up to 8 MPa) within appressorium to penetrate the plant cuticle layer (Howard et al., 1991; Howard and Valent, 1996; Talbot, 2003), and after penetration, the fungus develops bulbous biotrophic infectious hyphae in the rice leaf cells and typical necrotic lesions on the leaf surface (Kankanala et al., 2007). After 5–7 days, newly formed pyriform conidia differentiate from the hyphae on the lesion, and serve as inocula for secondary infection cycles (Talbot, 2003). These findings suggest that the sporulation and appressorium formation are essential for successful disease development. Thus, an understanding of the molecular mechanisms involved in these processes could provide insights into the nature of the plant–fungi interaction and is of great interest in the development of antifungal strategies.

Protein glycosylation is a post-translational modification conserved in organisms from yeasts to humans, and plays a critical role in determining the structure and function of numerous secreted and membrane-bound proteins (Lehle et al., 2006). In eukaryotic cells, there are two types of protein glycosylation (*N*- and *O*-glycosylation) that are highly regulated by the activity of protein- and site-specific enzymes (Fernández-Álvarez et al., 2009). *O*-mannosylation is a type of *O*-glycosylation that is characterized by the addition of mannose residues to target proteins, and is initially catalyzed by protein *O*-mannosyltransferases (PMTs), a family of proteins that are evolutionarily conserved from yeast to humans (Strahl-Bolsinger et al., 1999; Willer et al., 2003). In fungi, the PMTs are grouped into Pmt1, Pmt2, and Pmt4 subfamilies based on phylogeny, with each subfamily having three to seven members (Girrbach et al., 2000). In *Saccharomyces cerevisiae*, seven homologous PMT proteins have been identified and divided into the Pmt1, Pmt2, and Pmt4 subfamilies, which differ in their number of genes and the protein substrate specificity (Girrbach and Strahl, 2003). However, in many other filamentous fungi, including *M. oryzae*, only a single member in each Pmt subfamily was identified (Dean et al., 2005; Fernández-Álvarez et al., 2009; Goto et al., 2009; Mouyna et al., 2010; Gonzalez et al., 2013). In the past two decades, studies of the functions of fungal PMTs has been increasing, and now it is clear that protein glycosylation contributes to the modification of proteins involved in important processes, such as cell wall integrity (CWI), sensing of environmental signals, morphogenesis and the virulence of fungal pathogens (Gentzsch and Tanner, 1996; Prill et al., 2005; Olson et al., 2007; Zhou et al., 2007; Fernández-Álvarez et al., 2009, 2012). Simultaneous disruptions of three different types of *PMT* genes in *Schizosaccharomyces pombe* were lethal (Willer et al., 2005), suggesting that each class provided a unique function for *O*-mannosylation. In *S. cerevisiae*, the *PMT* genes are not individually essential for viability, probably as a result of gene redundancy (Gentzsch et al., 1995). Deletion of *PMT1* does not affect viability but leads to cells that tend to aggregate. Inactivation of both *PMT1* and *PMT2* causes defects in growth and resistance to antifungal drug (Lussier et al., 1995), whereas triple mutants are not viable, indicating that PMT protein activity is essential in *S. cerevisiae*, although individual genes are dispensable (Tanner et al., 1996; Loibl and Strahl, 2013). In *Candida albicans* and *Cryptococcus neoformans, Pmt* disruption affects morphogenesis and virulence (Prill et al., 2005; Olson et al., 2007). In filamentous fungus *Aspergillus fumigatus*, deletion of *Pmt4* results in abnormal growth, defective conidiation and associated proteomic changes, while disruption of *Pmt2* results in lethal growth (Mouyna et al., 2010). In *Aspergillus nidulans*, all the single *Pmt* disruption mutants were viable, but defective in cell wall integrity, hyphal growth and asexual development (Kriangkripipat and Momany, 2009). In *Ustilago maydis*, the three Pmt orthologs play diverse roles in fungal development. The deletion of *Pmt1* doesn't affect the fungal growth and plant infection, while the mutation in *Pmt2* is not viable, indicating an essential role in fungal development. By contrast, the disruption of *Pmt4* specifically affected appressorium formation, penetration and tumor formation in maize (Fernández-Álvarez et al., 2009). In *Botrytis cinerea*, PMTs are individually required for morphogenesis, fungal adherence, cell wall integrity and virulence on plants, and deletion of *PMT2* results in defects on the stability of the cell wall, poor sporulation and attenuated virulence on plants (Gonzalez et al., 2013). In *Beauveria bassiana*, PMTs play crucial roles on fungal development, and individual *Pmt* gene deletion results in defects on growth, conidiation, stress tolerance and virulence (Wang et al., 2014). In *Penicillium digitatum*, the disruption of *Pmt2* causes pleiotropic effects, including defects on cell wall integrity, conidiogenesis, virulence and resistance to the antifungal peptide PAF26 (Harries et al., 2015). Based on the above facts, it is therefore evident that the *O*-mannosyltransferases Pmts play important roles in fungal development and pathogenesis in pathogenic fungi.

Recently, evidence that the α-1, 3-mannosyltransferase ALG3 from *M. oryzae* play a critical role in mediating the glycosylation of secreted effectors, and thus required for fungal pathogenicity on host (Chen et al., 2014), suggest that protein glycosylation may be important for the pathogenic development of *M. oryzae*. However, till now, enzymes involving *O*-mannosylation pathway has not been characterized in the rice blast fungus. Here, we describe a detailed characterization of the role of *MoPmt2* in *M. oryzae*, and our results showed that *MoPmt2* contribute to fungal morphology, growth, CWI and virulence on host plants.

## Materials and methods

### Fungal strains and culture conditions

The *M. oryzae* Guy11 was used as wild-type strains throughout this work. Fungal mycelia grown in liquid complete media at 28°C for 2 days were harvested and used for genomic DNA and RNA extractions. For observing the mycelial growth, strains were inoculated in liquid CM as described in the reference (Guo et al., 2015). For conidiation, mycelial plugs were inoculated on RDC agar plates (Guo et al., 2011) and maintained at 28°C for 7 days in the dark followed 3–5 days constant fluorescent light condition to promote conidiation. For medium containing cell wall-perturbing agents, the final concentrations were 50, 100, 200 μg/mL for Congo red (CR, 860956, Sigma, China), and/or for Calcofluor white (CFW, F3543, Sigma, China), respectively. The inhibition rate was calculated by the method described in the reference (Guo et al., 2015).

### Yeast *Pmt2* mutant complementation

*S. cerevisiae* BY4741Δ*YAL023c* (Δ*Pmt2*) and the strain from which it was derived, BY4741 (*MATa*; *ura3*Δ*0*; *leu2*Δ*0*; *his3*Δ*1*; *met15*Δ*0*) were purchased from Euroscarf (http://www.uni-frankfurt.de/fb15/mikro/euroscarf/). The full-length of *MoPmt2* cDNA (XM_003715348.1) from *M. oryzae* was amplified using primer pairs Pmt2-YC1/ Pmt2-YC2. The PCR products, digested with *Hind*III and *Xho*I, were cloned into pYES2 (Invitrogen) and transformed into Δpmt2 mutant (BY4741, Δ*YAL023c::kanMX4*). Positive transformants were selected on SD medium lacking uracil. For complementation assays, all tested strains were grown and treated as described by Harries et al. (2015). The cells of the tested strains were diluted to an OD600 of 0.1 and 10 μL of ten-fold serial dilutions were spotted onto SC-Ura plates containing 2% glucose with and without 12.5 μg/mL CFW or 2% galactose with 12.5 μg/mL CFW, respectively, and grown for 3 days at 30°C. The wild type strain BY4741 as well as the *Pmt2* deletion mutant BY4741ΔYAL023c expressed empty pYES2 vector were used as a control.

### Multiple sequence alignment and phylogenetic analysis

The Pmt proteins from different organisms were obtained from NCBI database (http://www.ncbi.nlm.nih.gov/) using the BLAST algorithm (McGinnis and Madden, 2004). Sequence alignments were performed using the Clustal_W program (Thompson et al., 1994) and a phylogenetic tree was created with the Mega 4.0 Beta program (Tamura et al., 2007). Domain architecture was automatically provided by the SMART online software program (Letunic et al., 2012) or TMHMM Server v. 2.0 (Krogh et al., 2001).

### Gene disruption and complementation

The gene deletion mutants were generated using the standard one step gene replacement strategy. For gene replacement construct, DNA fragments of the 1.0 kb flanking regions of the target gene were amplified with the primers Pmt2-1F/Pmt2-1R and Pmt2-2F/ Pmt2-2R (Table S1), then a ~2-kb fragment containing the two flanking sequences was amplified by overlap PCR with primers Pmt2-1F / Pmt2-2R (Table S1), and then was inserted into the pMD19-T vector (Takara Co., Dalian, China) to generate plasmid pMDT–*MoPmt2*. The hygromycin B-resistance cassette, which was amplified with primers HPH-F/HPH-R by KOD-Plus-Neo (KOD-401, TOYOBO, Shanghai, China), was purified to insert into the *Pme*I site in plasmid pMDT–*MoPmt2* to generate deletion construct pMDT-*MoPmt2*-*hph*. A ~3.4-kb fragments, which was amplified from the deletion construct with primers Pmt2-1F/Pmt2-2R, was transformed into protoplasts of wild type Guy11. To obtain *MoPmt2* null mutants, all hygromycin B-resistant transformants were screened by PCR using the primer pairs P1/P2 and P3/P4 (Figure S1), respectively, and were further validated by Southern blot hybridization and RT-PCR (Figures S2D–F). For complementation of *MoPmt2*, the fragments containing the 1.5-kb native promoter region of the gene, *MoPmt2* full-length coding region and a 0.5-kb terminator sequence were amplified using primers Pmt2-C1/Pmt2-C2 and then inserted into the pMo1102 vector (unpublished plasmid) using the yeast gap repair approach (Park et al., 2004) to generate pMo1102::*MoPmt2*. The complemental fragment was then reintroduced into the *MoPmt2-6* mutant by the *Agrobacterium* mediated transformation of *M. oryzae*, and the complemented strains were validated by semiquantitative RT-PCR.

### Phenotypic characterization of mutants

To assess the growth rate, mycelia plugs of Guy11 and its derivative mutants were obtained from the edge of 5-day-old cultures and placed onto fresh media (CM, MM, OM, and RDC), followed by incubation in the dark condition at 28°C for 5 days (Guo et al., 2015). The conidia production of the tested strains was carried out by counting the number of conidia harvested with 5 ml of sterilized distilled water from 10-day-old RDC agar plates using a haemocytometer (Zhang et al., 2009). Conidium size was measured under a microscope as previously described (Guo et al., 2011). For conidium germination and appressorium formation, drops (20 μl) of conidial suspension (1.0 × 10^5^ spores/ml) were inoculated onto a hydrophobic coverslip and placed under humid conditions at 28°C. Conidia germination and appressoria formation were observed by microscopic examination of at least 99 conidia per replicate at each time point. Appressorium turgor was measured by incipient cytorrhysis assay using a 1-4 molar concentration of glycerol solution (Zhang et al., 2009). For conidium adhesion assay, drops (30 μl) of conidial suspension (1.0 × 10^5^ spores/ml) were placed on the hydrophobic surface of coverslips for 2 h in a humid box, then the coverslips were washed by pipetting 1 ml distilled water three times (each washing takes 3 s)., and the percentage of conidium adhesion was assessed as described previously (Han et al., 2015). All above experiments were independently repeated three times with three replicate each, and representative results from one experiment are shown in this study.

### Pathogenicity assay and infectious growth observation

For plant infection assays, conidial suspension (4 ml, 1.0 × 10^5^ spores/ml) containing gelatin (2%, wt/vol) were sprayed on 14-day-old rice seedlings (*Oryza sativa* cv CO-39) or drops of conidial suspension (10 μL, 1.0 × 10^5^ spores/ml) were placed on 7-day-old barley leaves (*Hordeum vulgare* cv Golden Promise). Inoculated plants were kept in a moist chamber in dark at 28°C for first 2 h, and then were moved to a moist chamber with a photoperiod of 12 h under fluorescent lights (Guo et al., 2011). The disease development on plants was assessed at 5 days after inoculation. For pathogenicity assay on abraded rice leaves, drops (10 μl) of conidia suspension (1.0 × 10^5^ ml^−1^) of the tested strains were placed on wounded rice leaves and kept in a moist chamber as described above, and their virulence was evaluated at 5 days after incubation. Conidiation on the lesion was treated as described above and observed 15 days after incubation. Plant penetration assays were carried out using 7-day-old barley leaves (Chen et al., 2014). Conidial suspension (1 × 10^4^ spores/ml) was dropped onto barley leaves and incubated at 28°C in a moistened chamber. Invasive growth inside plant cells was examined after at 48 and 72 h using a light microscopy. These experiments were repeated three times, and representative results from one experiment are shown.

### FITC-ConA and CFW staining

FITC staining was performed with conidia collected from RDC agar plates (Zhang et al., 2009). Conidia suspension (1 × 10^5^ conidia ml^−1^) was inoculated on the hydrophobic surface of coverslips and then treated as described in reference (Hamer et al., 1988) at 0.5 and 24 h, respectively. Microscopic examination of stained conidia and mature appressorium (>100 conidia or appressorium) was carried out using a Nikon inverted T*i*-S epifluorescence microscope (Nikon Co., Tokyo, Japan). For CFW staining, conidia suspension (1 × 10^4^ conidia ml^−1^) was inoculated on glassslides covered with a thin layer of water agar and kept in a moist plate for 48 h or on hydrophobic coverslips and kept in a moist plate for 24 h, then both the vegetative hyphae and appressorium stained by CFW was observed using the Nikon inverted T*i*-S epifluorescence microscope (Nikon Co., Tokyo, Japan), respectively.

### Nucleic acid manipulation and Southern blotting

The standard method was used to prepare genomic DNA for both diagnostic PCR and Southern blot hybridization (Sambrook et al., 1989). A DNA hybridization probe, which is amplified by primer set Pmt2-P1/Pmt2-P2, were labeled with digoxigenin-11-dUTP using DIG-High prime according to the manufacturer's instructions (11745832910, Roche, China). Southern hybridization experiments, including restriction enzyme digestion, DNA gel blotting and hybridization were performed as described by Guo et al. (2015). Total RNA samples from mycelia, conidia and plants infectious stages (8, 24, 48, and 72 h) were extracted using the methods described in E.Z.N.A. Total RNA Kit I (R6834-01, Omega Bio-Tek, Norcross, USA).

### qRT–PCR, RT–PCR, and gene expression analysis

For RT-PCR and qRT-PCR, 5 μg of total RNA were reversely transcribed into first-strand cDNA using the ReverTra Ace^®;^ qPCR-RT Master Mix (FSQ-301, TOYOBO, Shanghai, China). Confirmation of deletions and reintroduction of *MoPmt2* gene was made with primer pairs Pmt2-S1/Pmt2-S2 (Table S2). The stable expression *actin* gene (*MGG_03982.5*) and β-*tubulin* gene (*MGG_00604.5*) amplified by primer pairs Actin-F/Actin-R and β-tubF/β-tubR, respectively, (Table S2) was used as internal control (Guo et al., 2015).

qRT-PCR were performed using a BIO-RAD CFX96 touch q-PCR system (BIO-RAD, Hercules, California, USA), following previously established procedures: 3 min at 95°C (one cycle) followed by 15 s at 95°C, 30 s at 60°C, and 30 s at 72°C (40 cycles). To measure the relative abundance of *MoPmt2* transcripts, the average threshold cycle (Ct) was normalized to that of *actin* gene (*MGG_03982.5*) and expressed as 2^−ΔCt^, where −ΔCt = (C_t, target gene_ − C_t, actin gene_). Fold changes in expression during fungal development and infectious growth were calculated as 2^−ΔΔCt^, where −ΔΔCt = (C_*t*, target gene_ − C_t, actin gene_)_testcondition_ − (C_t, target gene_ − C_t, actin gene_)_CM_ (Livak and Schmittgen, 2001). Three independent pools of tissues in three sets of experimental replicates were performed and primer pairs used in this section were listed in Table S2.

### Measurement of enzyme activity in extracellular culture filtrate

Enzyme activity was assayed using culture filtrate from 2-day old CM liquid culture. For measurement of peroxidase and laccase activity, a reaction mixture (1 ml) containing 50 mM acetate buffer (pH 5.0) and 10 mM 2, 2′- azino-di-3-ethylbenzthiazoline-6-sulphonate (ABTS, Sigma, A1888) was mixed with the culture filtrate (200 μl) and incubated at 25°C for 5 min with or without 3 mM of H_2_O_2_. Absorbance was evaluated at 420 nm.

### Transmission electron microscopy

Transmission electron microscopy was carried out using hyphae grown in liquid CM for 48 h. The hyphae were fixed in 2.5% (v/v) glutaraldehyde and 1% (v/v) osmium tetroxide. Sections were prepared and visualized using a H-7650 transmission electron microscope (Hitachi, Tokyo, Japan) as described by Xu et al. (2010).

## Results

### Identification of three *Pmt* genes in *M. oryzae*

**Figure 1:**
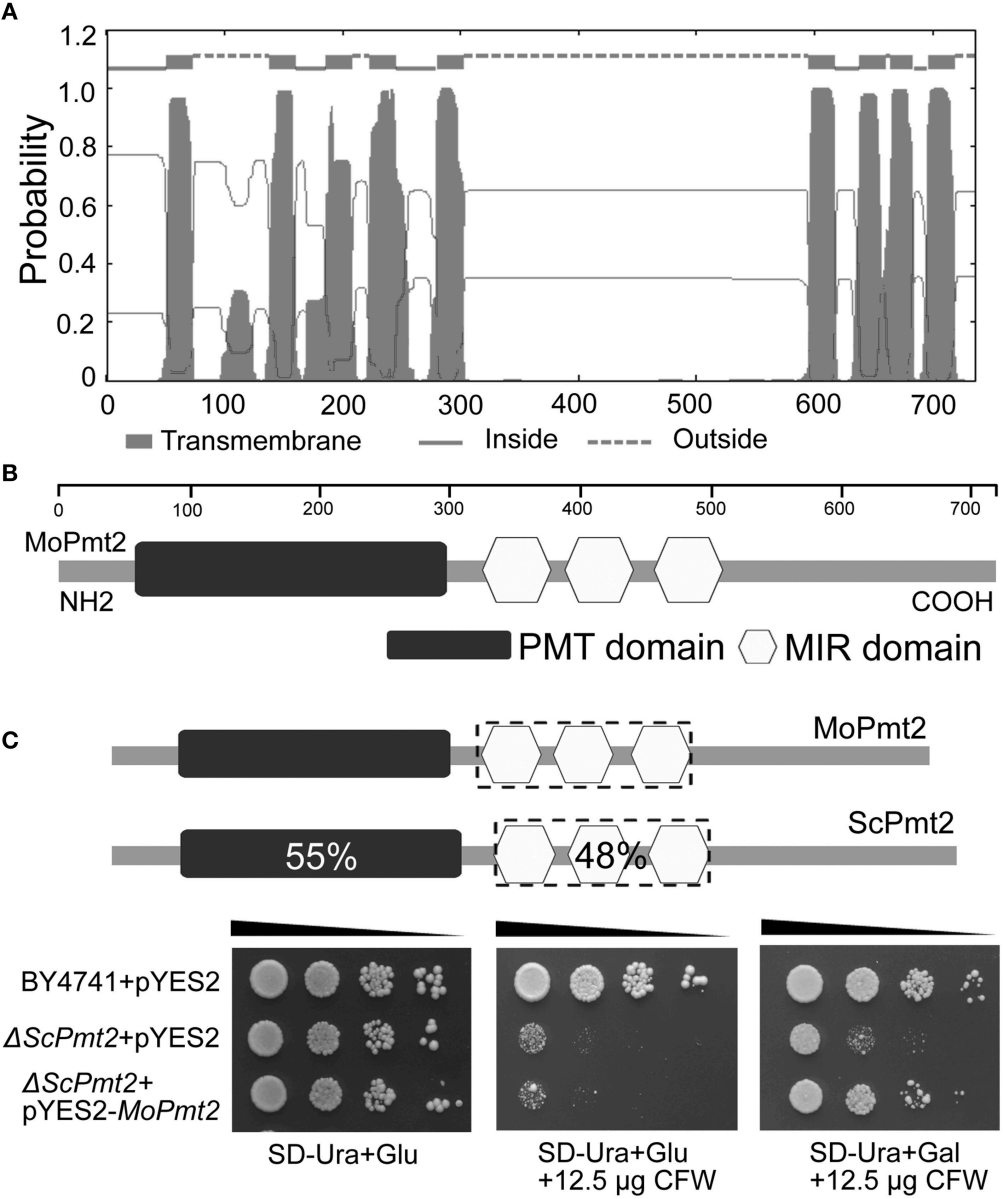
***M. oryzae* MoPmt2 encodes a functional homolog of *S. cerevisiae* Pmt2. (A)** Prediction of transmembrane structure in MoPmt2. The position of each transmembrane domain was generated by the TMHMM Server v. 2.0 online program, and the MoPmt2 possess the typical hydropathy profiles of *O*-mannosyltransferases. **(B)** Conservation of the Pmt sequence motifs in MoPmt2. Physical map of MoPmt2 revealed two conserved motifs (Pmt and MIR) of the PMT family at the N-terminal transmembrane region and central hydrophilic region. **(C)** Functional complementation of *S. cerevisiae Pmt2* mutant. Serial 10-fold dilutions of cells from wild-type strain BY4741 transformed with pYES2 and the *Pmt2* strain transformed with either pYES2 or pYES2-*M*oPmt2 are dotted onto SC-Ura plates with Glu or Gal supplemented with 12.5 μg/mL CFW as indicated, and incubated at 30°C for 72 h.

In our previous study, the *Moap1* mutants were identified as non-pathogenic to rice plants (Guo et al., 2011). To investigate the possible reason for this, a serial analysis of gene expression (SAGE) libraries for the wild type Guy11 and *Moap1* mutant were generated. In the SAGE libraries, three *O*-Mannosyltransferases encoding genes were identified with different expression patterns, with MGG_02954 and MGG_04427 showing 0.35- and 1.0-fold increased expression, respectively, while MGG_07190 showing 1.35-fold reduced expression in the *Moap1* mutants. Sequence alignment using amino acid sequences of the three *O*-Mannosyltransferases revealed the MGG_02954, a protein of 998 amino acids, showed an amino acid identity of 39% to *S. cerevisiae* Pmt1p; MGG_07190, of 737 amino acids, showed 47% identity to *S. cerevisiae* Pmt2p; and MGG_04427, of 775 amino acids, showed 45% identity to *S. cerevisiae* Pmt4p (Table S1; Gentzsch and Tanner, 1996). The hydropathy analysis of *M. oryzae* Pmt sequences revealed the presence of two large transmembrane domains, located in their N- and C-terminal regions and separated by a central hydrophilic region (Strahl-Bolsinger and Scheinost, 1999; Figure 1A). In addition, analysis of the candidate proteins showed the presence of two characteristic domains of the PMT family: the PMT domain, a region implicated in PMT complex formation and *O*-mannosyltransferase activity, found within the N-terminal transmembrane region, and the mannosyltransferase IP3 ryanodine receptor domain (MIR) composed of three submotifs, located in the hydrophilic central region (Girrbach et al., 2000; Figure 1B; Table S1). The assignment of these identified genes as protein *O*-mannosyltransferases was further confirmed by phylogenetic reconstruction with homologous fungal genes, and they were grouped into each PMT subfamily (Lehle et al., 2006; Figure S1). Thus, consistent with their hydropathy profiles, conserved sequence motifs, and our phylogenetic analysis, we can provisionally named MGG_02954, MGG_07190 and MGG_04427 as MoPmt1, MoPmt2, and MoPmt4 respectively.

### MoPmt2 encodes protein *O*-mannosyltransferases

The SAGE libraries revealed that *MoPmt2* showed transcriptional reduction in the *Moap1* mutant, indicating its potential roles in regulating fungal development and pathogenicity in *M. oryzae*. Therefore, the functional roles of *MoPmt2* were characterized in this study. In *S. cerevisiae*, the *pmt2* gene is responsible for cell wall integrity, and Δ*pmt2* mutant has defects in cell wall and exhibit increased sensitivity to the chitin-binding fluorophore CFW (Harries et al., 2013). To provide experimental evidence that *MoPmt2* encodes a functional homolog of *S. cerevisiae* Pmt2 with *O*-mannosyltransferase activity, we tested the ability of *MoPmt2* to complement a yeast strain lacking *pmt2*. Our results showed that the *S. cerevisiae* Δ*pmt2* mutants transformed with the expression vector pYES2-*MoPmt2* restored the resistance to CFW under induction conditions (Figure 1C), indicating that *M. oryzae* Pmt2 is functional homolog of the *S. cerevisiae* Pmt2. Moreover, it also confirms our phylogenetic-based assignment of PMT family members.

### The expression and disruption of *MoPmt2* gene in *M. oryzae*

To gain insight into the possible function of MoPmt2 in *M. oryzae*, the changes in *MoPmt2* gene expression were analyzed by quantitative RT-PCR (qRT-PCR), and found that *MoPmt2* gene is expressed during fungal vegetative growth and plant infection, with the highest induction at conidia stage (7.4-fold; Figure S2A), compared with the stable expression of *actin* gene.

**Figure 2:**
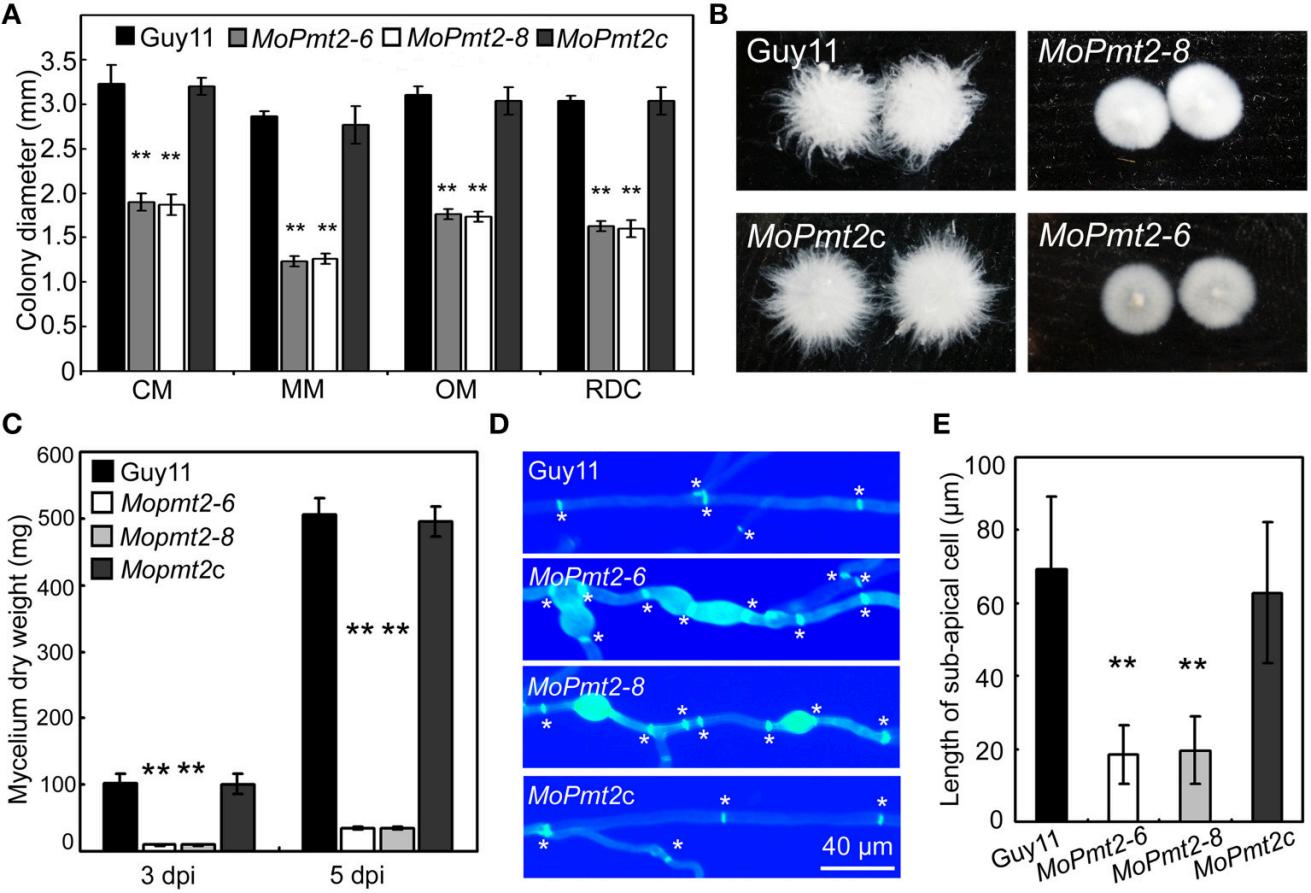
**The effect of MoPmt2 on mycelia growth of *M. oryzae*. (A)** Mycelial growth is altered in the *MoPmt2* mutant. Colony diameters of the tested strains on different media were measured and statistically analyzed by Duncan analysis. **(B)** Phenotype of mycelia grown in liquid CM. All tested strains were inoculated in liquid CM for 48 h at 28°C in darkness and then photographed. **(C)** Measurement of fungal biomass. The fungal mycelia of the tested strains, grown in liquid CM for 3 and 5 days respectively, were collected and freezing dried, and were statistically analyzed. **(D)** Mycelia growth on water agar media. CFW staining of mycelia is used to show the distance of septa and swollen of mycelia. Asterisks in white color indicate septa. **(E)** The average length of the sub-apical cell of vegetative hyphae in Guy11, *MoPmt2-6, MoPmt2-8*, and *MoPmt2c*. The mean and standard deviations were calculated based on three independent experiments by measuring at least 50 sub-apical cells in each replicate. Error bar represents standard deviation, and asterisks in this figure indicate significant differences from the control (*P* < 0.01).

**Figure 3:**
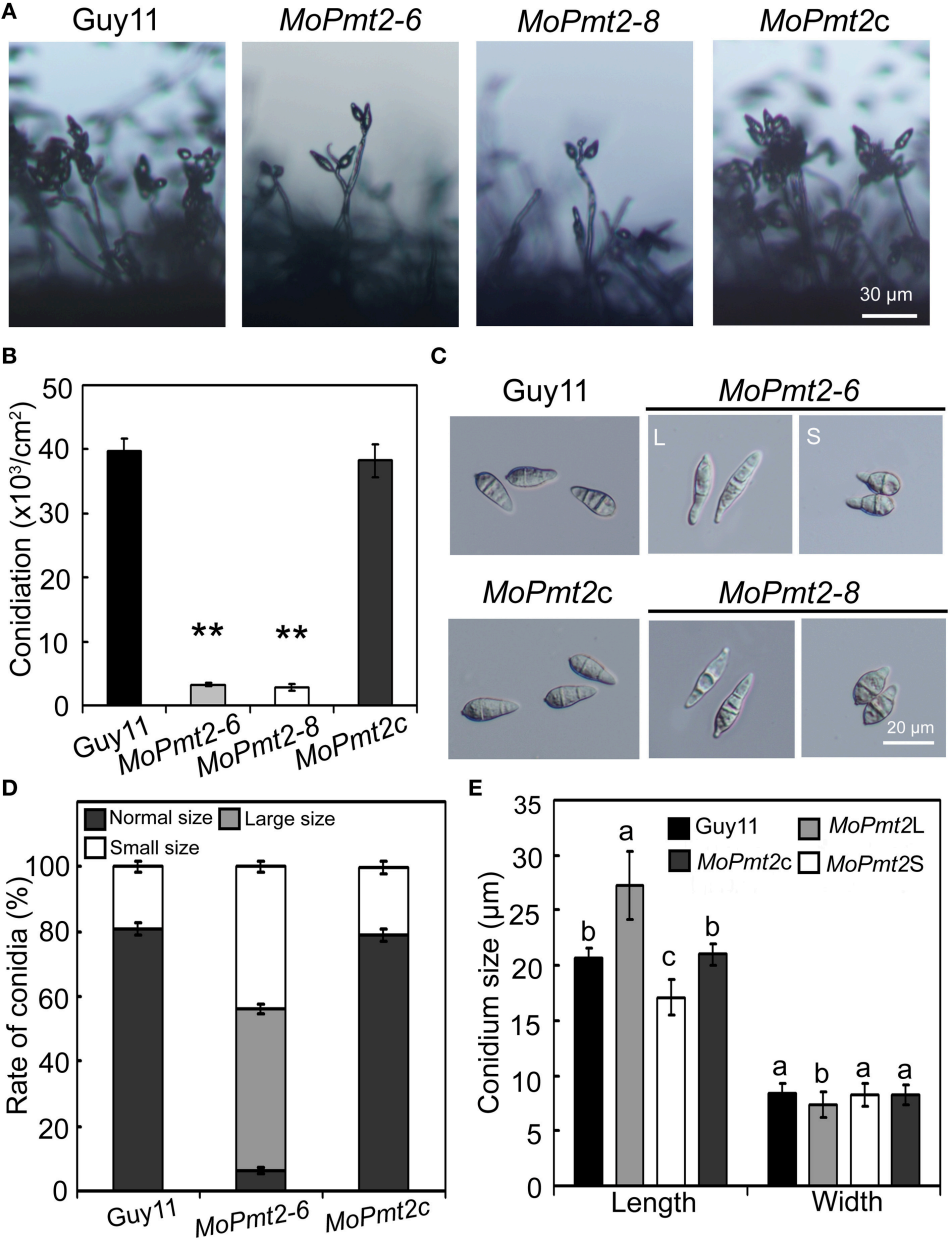
**The effect of MoPmt2 on conidiation and conidial morphology of *M. oryzae*. (A)** *MoPmt2* deletion results in defects on conidiation. The development of conidia on conidiophores was examined by light microscope using strains grown on RDC medium for 7 days. Scale bar = 30 μm. **(B)** Statistical analysis of conidiation. The conidia produced by the tested strains grown on RDC medium for 14 days were measured and analyzed by Duncan analysis (*p* < 0.01). Asterisks indicate significant differences of conidiation among tested strains. Error bar represents standard deviation. **(C)** Conidial morphology comparison. Conidia of tested strains were collected from 14-day-old cultures, and then photographed under light microscope. Bars = 20 μm. **(D)** The percentage of each type of abnormal conidia. Conidia of tested strains were harvested from 14-day-old cultures, and the rate of large and small conidia was calculated and statistically analyzed, respectively. **(E)** Conidia sizes comparison. The conidia sizes were determined as width by length from 297 conidia of each strain.

**Figure 4:**
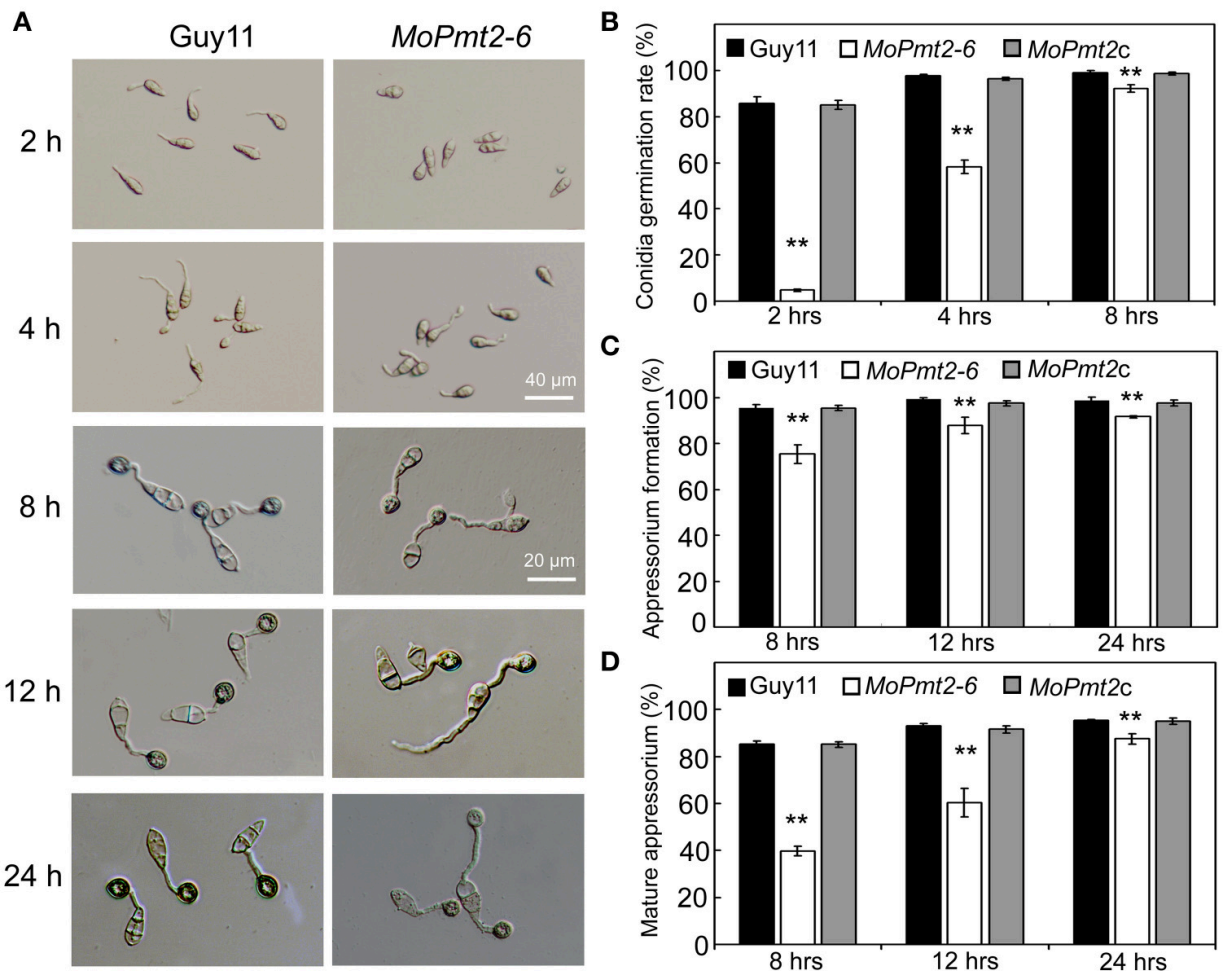
**Conidial germination and appressorium formation. (A)** Delayed conidial germination and appressorium formation of the *MoPmt2* mutant. Conidia of indicated strains were harvested from 14-day-old cultures. Droplets of conidial suspension (1 × 10^5^ ml^−1^) were inoculated on the hydrophobic surface of the coverslips for indicated time, and then photographed. Scale bars are indicated in the figure. **(B)** Statistical analysis of conidial germination. Germinated conidia were counted at each indicated time under a light microscope, and statistically analyzed by Duncan analysis. **(C)** Statistical analysis of appressorium formation. Appressorium formed at the germ tube were counted and statistically analyzed. **(D)** Measurement of mature appressorium. Appressorium formed at the germ tube with melanin were counted, and the ratio of mature appressorium was statistically analyzed. Asterisks in this figure indicate significant differences from the control (*p* < 0.01). Error bar represents standard deviation.

**Figure 5:**
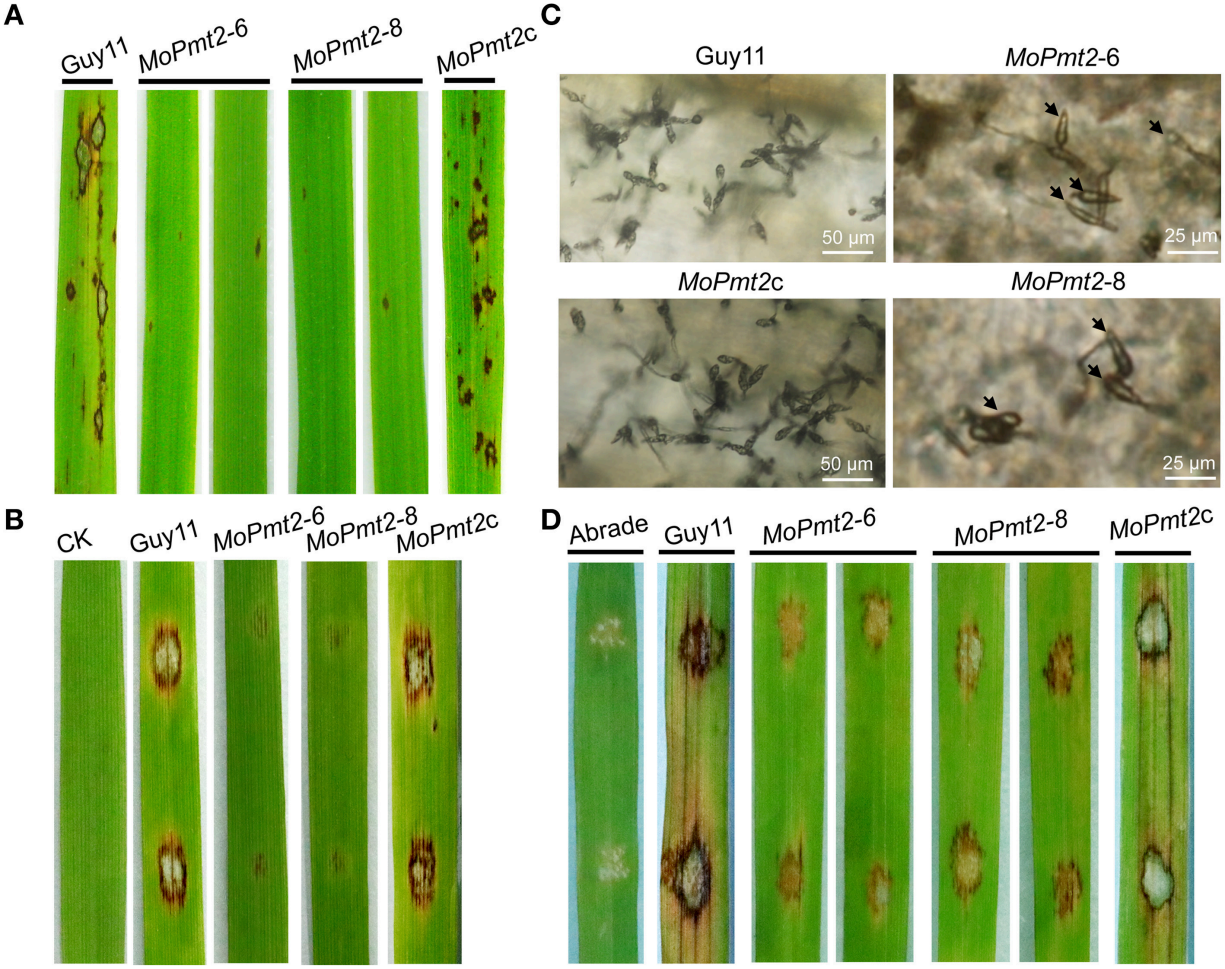
**Pathogenicity assays. (A)** The pathogenic development of the mutants on rice leaves. The *MoPmt2* deletion attenuated pathogenicity on rice leaves. Diseased leaves were harvested 5 days after inoculation. **(B)** Pathogenicity assays on barley leaves. The *MoPmt2* deletion attenuated virulence on barley leaves. Diseased leaves were harvested 5 days after inoculation. **(C)** Microscopic examination of conidia produced on the lesions. Diseased leaves were harvested 15 days after inoculation and observed under a light microscope. **(D)** Pathogenicity assay on abraded rice leaves. Drops (10 μL) of conidia suspension from tested strains were inoculated on abraded rice leaves and a virulence defect was indicated in the mutants, compared with the control strains at 5 dpi.

**Figure 6:**
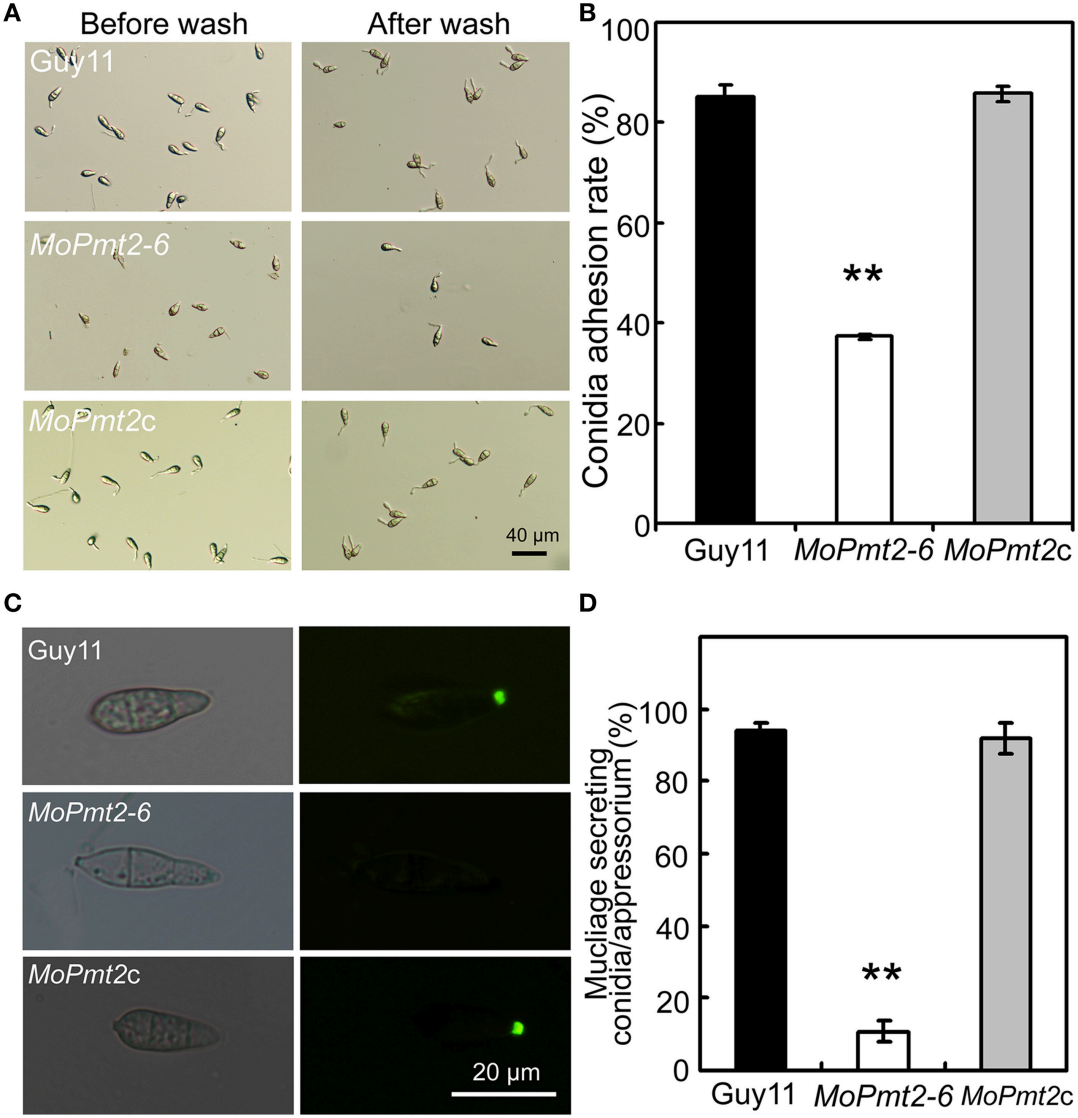
**The effect of MoPmt2 on conidia adhesion. (A)** Inability of *MoPmt2* conidia to adhere to a hydrophobic surface. Conidia suspension of tested strains grew on a hydrophobic coverslips for 2 h was washed with sterilized water by pipetting and then visualized with brightfield optics of Nikon inverted T*i*-S microscope. Scale bar = 40 μm. **(B)** Statistical analysis of conidia adhesion to hydrophobic coverslips. The numbers of conidia adhesive to the coverslips after wash were counted and statistically analyzed by Duncan's analysis (*p* < 0.01). Asterisks indicate significant differences of conidia adhesion among strains. Error bar represents standard deviation. **(C)** STM secretion test. STM from the conidia of Guy11, *MoPmt2* mutant and *MoPmt2c* were stained with FITC-ConA, and the fluorescence signal from the germinated conidia were visualized by a Nikon inverted T*i*-S epifluorescence microscope. **(D)** Statistical analysis of conidia stained by FITC-ConA. The numbers of conidia stained by FITC-ConA were counted and statistically analyzed by Duncan's analysis (*p* < 0.01). Asterisks indicate significant differences among strains. Error bar represents standard deviation.

**Figure 7:**
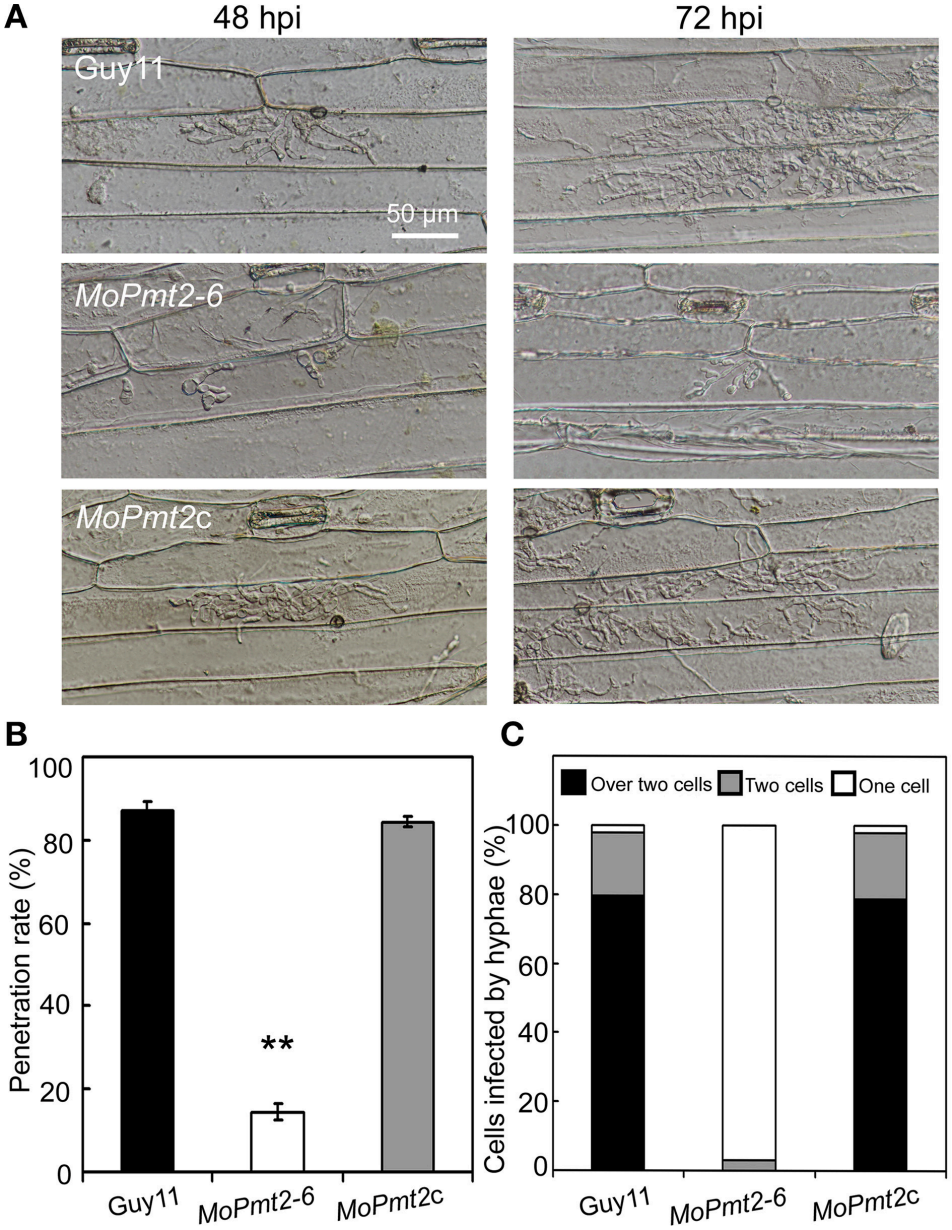
**The deletion of *MoPmt2* results in impaired appressorium penetration. (A)** The *MoPmt2* mutants are defective on appressorium-mediated penetration. The *MoPmt2* mutants exhibited penetration defects on barley leaves after 48 and 72 h, respectively, in contrast with control strains. Scale bar = 50 μm. **(B)** Statistical analysis of appressorium penetrated in host cells. The appressoria that penetrated into barley epidermal cells were counted at 48 hpi, and the data were statistically analyzed. Asterisks indicate significant differences among strains. Error bar represents standard deviation. **(C)** Quantification of hyphae infection on barley epidermal cells. The percentage of infected cells occupied by infectious hyphae of tested strains at 72 hpi were measured and statistically analyzed.

**Figure 8:**
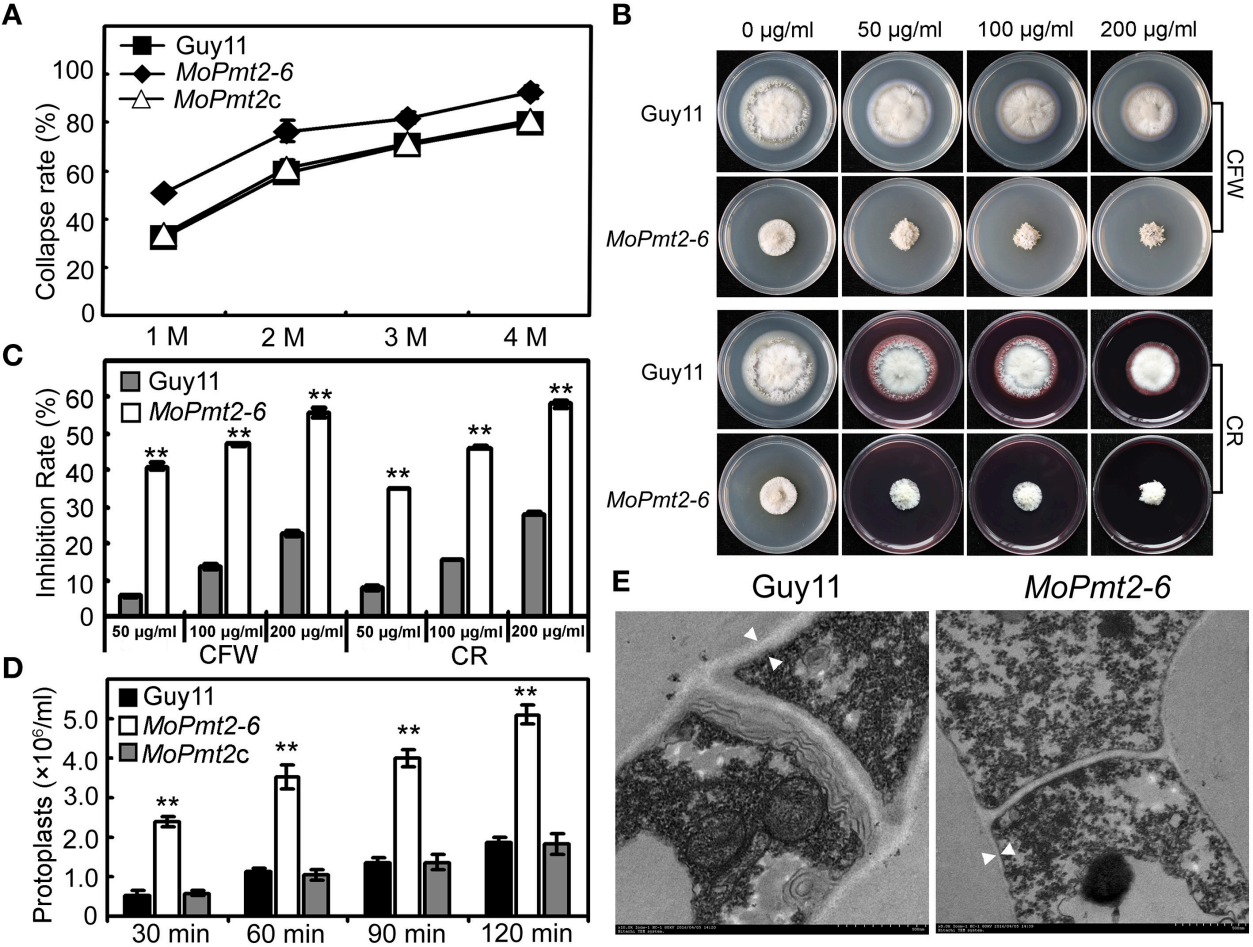
**The deletion of *MoPmt2* impaired cell wall integrity of *M. oryzae*. (A)** Measurement of collapsed appressoria. The collapsed appressoria (>100) were observed at each glycerol concentration and statistically analyzed. **(B)** Sensitivity of *MoPmt2* mutants to cell wall damaging agents. All tested strains were inoculated on CM containing cell wall-perturbing agents (CR and CFW) with final concentrations of 50, 100, 200 mg/mL, respectively. **(C)** Statistical analysis of mycelial growth of the tested strains under CR and CFW. **(D)** Protoplast release assay. Protoplasts released under the treatment of lysing enzymes were counted and statistically analyzed at each indicated time. Asterisks in this figure indicate significant differences among the strains (*p* < 0.01). Error bar represents standard deviation. **(E)** Ultrastructure of the cell wall in the *MoPmt2* mutant. Cell wall ultrastructures of mycelia of wild type strain Guy11 and the *MoPmt2* mutant were observed by sectioning and TEM. Distances between the white triangles indicated the width of cell wall. Three independent experiments were carried out and representative images from one experiment were shown.

We generated *MoPmt2* mutants using the *MoPmt2* deletion vector which contains the hygromycin resistance gene (Figure S2B). The transformants that confers resistance to the antibiotic hygromycin B were verified by diagnostic PCR (**Figure S2C**). The putative mutants were further confirmed by Southern blot and semiquantitative RT-PCR (RT-PCR) analysis (Figures S2D–F), and two deletion mutants, *MoPmt2-6* and *MoPmt2-8*, were obtained for further analysis. To ascertain that the observed phenotypes of the *MoPmt2* mutants are caused by the deletion of *MoPmt2*, a complemented strain *MoPmt2c* was generated and verified by RT-PCR (Figure S2F). As expected, the complemented strain recovered all the defects described in this study (Figures 2–**8**).

### *MoPmt2* deletion leads to retarded vegetative growth

To investigate the role of *MoPmt2* on mycelium growth in *M. oryzae*, mycelial growth of wild type strain Guy11, the *MoPmt2* mutants and complemented strain *MoPmt2c* was compared on complete media (CM), minimal media (MM), oatmeal medium (OM), and RDC medium (Guo et al., 2015). Mycelial growth of the *MoPmt2* mutants differed from that of Guy11, with a significant reduction in radial growth on the four media, compared to Guy11 and *MoPmt2c* (Figure 2A; Figure S3). In liquid CM media, we found that mycelial growth of the *MoPmt2* mutants was more compact than that of Guy11 and *MoPmt2c* (Figure 2B), and a subsequent assay indicated significantly less fungal biomass for the *MoPmt2* mutants than for Guy11 and *MoPmt2c* after incubating in liquid CM for 72 and 120 h, respectively (Figure 2C). In view of these phenotypes, we examined the morphology of *MoPmt2* mutants by microscopy after CFW staining. Our results showed that *MoPmt2* mutants presented more septa, which were intensively stained and had shorter interseptal distances than those of Guy11 and complemented strain *MoPmt2c* (Figures 2D,E). In addition, The *MoPmt2* mutants also showed defects in polarity, with more branching hyphae and globular balloon-like structures (Figure 2D; Figure S4A).

### *MoPmt2* plays critical role in asexual spore development

It is clear that asexual spores play an important role in the disease cycle of *M. oryzae* (Lee et al., 2006), thus, sporulation of the Guy11, *MoPmt2* mutants (*MoPmt2-6* and *MoPmt2-8*), and the complemented strain *MoPmt2c* was compared on 14 day old RDC cultures. Our findings revealed that conidiation was dramatically reduced by approximately 12 to 14-fold in *MoPmt2* deletion mutants (*MoPmt2-6*, 12-fold; *MoPmt2-8*, 14-fold), compared with Guy11 and complemented strains *MoPmt2c* (Figures 3A,B). In addition, of the spores that formed in *MoPmt2-6*, most exhibited abnormal, with 49.9% in large size and 43.7% in small size, compared with Guy11 and *MoPmt2c* (Figures 3C–E; Figure S5). Combined with gene expression profiles that *MoPmt2* showed a much higher level of expression in conidia (Figure S2A), we concluded that MoPmt2 plays an important role in conidial development.

### *MoPmt2* is responsible for conidia germination and appressorium formation

Conidia germination is the vital step during *M. oryzae* infection (Howard et al., 1991). Therefore, we measured the germination ability of conidia on a hydrophobic surface of coverslips. Our results showed that conidial germination was significantly delayed in the *MoPmt2* mutant when compared with that of Guy11 and *MoPmt2c*. By 2 h, only 4.7% of *MoPmt2* conidia germinated compared with 85.9% of the Guy11 and 85% of the *MoPmt2c*. When prolonged the inoculation time to 8 h, even though the conidial germination of the mutant gradually increased to 92.3%, it was still significantly less germination by the mutant than that of by Guy11 (Figures 4A,B).

The appressorium is a typical penetration structure that enables the *M. oryzae* to invade into host cells (Howard and Valent, 1996). Thus, we also evaluated the ability of appressorium formation by the mutants, and found that appressoria developed by the *MoPmt2* mutants were 75.4% by 8 h, 87.9% by 12 h, and 91.6% by 24 h, with a dramatic decrease compared with that of by Guy11 and *MoPmt2c* (Figures 4A,C). Meanwhile, of those appressoria formed by the mutant at each time point, only 39.7, 60.3, and 87.4% were melanic and non-reduced in size, compared with 85.2, 92.9, and 95.3% of that by Guy11 (Figure 4D). In addition, we also find that over 53% of the conidia were bipolar germination, compared with less than 8% of that by Guy11 and *MoPmt2c* strain at 24 hpi (Figure 4A; Figures S4B,C). Based on the above, we conclude that the *MoPmt2* is associated with both conidia germination and appressorium development in *M. oryzae*.

### MoPmt2 is essential for pathogenic development

To investigate the role of *MoPmt2* in pathogenic development, conidial suspension (1 × 10^5^ conidia ml^−1^) of the tested strains were inoculated on 14-day-old susceptible rice seedlings (*Oryza sativa* cv CO-39) and/or 7-day-old barley leaves by spraying. The wild type strain Guy11 caused numerous typical necrotic lesions, whereas the *MoPmt2* mutants developed small and restricted lesions on host plants (Figures 5A,B). Meanwhile, most of those lesions caused by the *MoPmt2* mutants seldom produced conidia, compared to wild type Guy11 and *MoPmt2c*. When the incubation time was extending to 15 days after inoculation, only few conidia were observed on the larger lesions (Figure 5C). To further determine the role of *MoPmt2* in pathogenicity, conidial suspension of the tested strains was inoculated on wounded rice leaves. The results revealed that the wild type Guy11 were fully pathogenic, and developed extendible and necrotic lesions, in contrast, the *MoPmt2* mutant only causes small lesions on the inoculation sites (Figure 5D). When the *MoPmt2* gene was reintroduced in the mutant, it recovered the pathogenicity on both rice and barley plants (Figures 5A–D), suggesting a potential role for the MoPmt2 in plant invasion and colonization.

### *MoPmt2* deletion results in defects on fungal adhesion

In *M. oryzae*, the persistent adhesion of the three-celled conidia to the rice leaf by means of the spore tip mucilage is a prerequisite for pathogenic development (Hamer et al., 1988). Thus, to identify the reason for attenuated virulence of *MoPmt2* mutants, we firstly tested conidium adhesion to a hydrophobic surface due to the fact that the spore tip mucilage could stick to hydrophobic coverslips (Hamer et al., 1988; Han et al., 2015). Our results showed that more than 63% of conidia of the *MoPmt2* mutant were washed away from the hydrophobic surface of coverslips, whereas only 15% of those by the wild type and *MoPmt2c* (Figures 6A,B), indicating that *MoPmt2* plays a critical role in fungal adhesion in *M. oryzae*. As the lectin concanavalin A (ConA) conjugated to fluorescein isothiocyanate (FITC) could be used for detecting STM (Hamer et al., 1988), thus, STM secreted by Guy11, *MoPmt2* mutant and complemental strain *MoPmt2c* were compared on hydrophobic glass cover slips. At 0.5 hpi, conidia of Guy11 showed a very strong signal for STM compared with those of *MoPmt2* mutant, with 93.9 and 91.8% conidia from Guy11 and *MoPmt2c* strain, respectively, showing fluorescence, in contrast to 10.9% conidia from *MoPmt2* mutant (Figures 6C,D). When the incubation time was prolonged to 24 h, however, no obvious differences were observed by comparison of fluorescence signal from mature appressoria of Guy11 and *MoPmt2* mutant (Figure S6A).

### MoPmt2 is required for penetration and invasive hyphae growth

To further understand the role of *MoPmt2* in disease development, we carried out penetration assays on the barley leaves (Figure 7A). The appressoria formed by *MoPmt2* mutants could less effectively penetrate into the epidermal cells of barley leaves at 48 hpi when compared to Guy11 and *MoPmt2c*. In addition, most of the invasive hyphae developed by the penetrated appressorium of the mutants displayed extremely retarded growth in the host cells (Figure 7A). When infected leaves were examined after 72 hpi, most invasive hyphae of *MoPmt2* mutant had still failed to colonize the leaf epidermis beyond the first cell (Figure 7B). In contrast, the wild type and *MoPmt2c* could freely expand and successfully colonized new cells across the cell walls of the initially invaded cell (Figures 7A,C). These results indicated that MoPmt2 is required for appressorium-mediated penetration as well as invasive hyphae growth in host cells, and the defect on these aspects might be responsible, at least in part, for the reduction of pathogenicity.

### MoPmt2 is required for turgor generation and cell wall integrity

In *M. oryzae*, the defects in cell wall composition can affect the accumulation of turgor pressure and impair the successful infection of rice plants (Howard et al., 1991; Howard and Valent, 1996; Thines et al., 2000). Based on penetration-defective phenotypes of the *MoPmt2* mutants, appressorium turgor pressure of the *MoPmt2* mutant was compared to wild-type Guy11, and the results showed that appressoria of the *MoPmt2* mutant were in a tendency to collapse at lower glycerol concentrations, compared to those of by the Guy11 and *MoPmt2c* (Figure 8A), suggesting a defect in maintaining appressorium cell wall integrity in *MoPmt2* mutant. In M. oryzae, an intact cell wall is the guarantee of full virulence on rice plants (Jeon et al., 2008), we thus examined the sensitivity of the transformants to different cell wall inhibitor, and the results showed that *MoPmt2* mutants showed significantly increased sensitivity to cell wall damaging agents CFW and CR, respectively (Figures 8B,C). Meanwhile, CFW staining of appressorium also revealed that much stronger fluorescence signal was observed around the appressorium of Guy11 and *MoPmt2c*, compared to *MoPmt2* mutant (Figure S6B), indicating less chitin accumulated on the cell wall of *MoPmt2* mutant. Moreover, protoplast release by the *MoPmt2* mutant was much faster than that of Guy11 and *MoPmt2c* after enzyme treatment for 30, 60, 90, and 120 min (Figure 8D), suggesting that cell wall integrity was impaired in the *MoPmt2* mutants. TEM assay further confirmed this phenotype and the cell wall ultrastructures of *MoPmt2* mutants were much thinner than wild type Guy11 (Figure 8E), indicating CWI defect may be results in failure of accumulation of appressorium turgor pressure, and thus attenuation of pathogenicity on rice plants.

### *MoPmt2* deletion attenuates the activity of extracellular peroxidases and laccases

In *M. oryzae*, the secreted extracellular peroxidases were presumed to be responsible for CR degradation, which, as a result, could generate a bright halo around the colony (Guo et al., 2011). In the examination of cell wall integrity of the *MoPmt2* mutants, we found that the degradation halo of CR by the *MoPmt2* mutants was not as apparent as the wild type (Figure 8B), indicating a deficiency of the CR-degrading activity in the *MoPmt2* mutants. An enzyme activity assay using ABTS as substrate revealed that the *MoPmt2* mutant severely reduced its peroxidase activity in the extracellular culture filtrate (Figure S7A). In addition, the activity of additional extracellular enzyme laccases was determined and found that the decreased laccase activity was also observed in the *MoPmt2* mutant, with lower levels of laccase activity in the culture filtrate, compared with the wild-type strain (Figure S7B).

## Discussion

In this study, three putative *O*-mannosyltransferases, with one member in each Pmt subfamily, were identified in rice blast fungus *M. oryzae*, which is consistent with findings that showed the existence of only one ortholog for each Pmt subfamily in other filamentous fungi and in the fission yeast (Willer et al., 2005; Fernández-Álvarez et al., 2009; Goto et al., 2009; Gonzalez et al., 2013; Harries et al., 2015). Amino acid sequence alignment showed that, similar to other members of the Pmt2 family in fungi, MoPmt2 contains a conserved the PMT domain in the N-terminal transmembrane region, and a MIR domain in the hydrophilic central region, which share 55 and 48% amino acid identity to the respective domains of Pmt2p from *S. cerevisiae* (Gentzsch and Tanner, 1996). Compared to *MoPmt1* and *MoPmt4*, the *MoPmt2* showed reduced transcriptional expression in the *Moap1* mutant, indicating distinctive roles during fungal development and pathogenicity. The functional analysis by complementation of yeast *Pmt2* deletion mutant demonstrated that MoPmt2 is homologous to Pmt2 from *S. cerevisiae*. These findings are also consistent with previous findings in *P. digitatum*, and suggest a conserved role of MoPmt2 in the regulation of growth and development in *M. oryzae* (Harries et al., 2015).

Previous studies revealed that the *O*-mannosyltransferase encoding gene *Pmt2* had diverse roles in fungal development (Fernández-Álvarez et al., 2009; Goto et al., 2009; Fang et al., 2010; Shimizu et al., 2014; Wang et al., 2014). In *M. oryzae*, our findings reveal that the *MoPmt2* deletion mutants are viable, but show severe defects in polarity, with more branching hyphae and septa, and globular balloon-like structures as compared to Guy11, which is similar to previous findings in *P. digitatum* and *B. cinerea* (Gonzalez et al., 2013; Harries et al., 2015). Previous studies in null *chs* mutants of *P. digitatum* revealed that the globular structures are resulted from abnormal fungal cell wall synthesis (Gandia et al., 2014). We therefore stained the cell wall of mycelia with CFW and found that these morphological defects are ascribed to production of cell enlargements with altered chitin content (Figure 2D), similar to results identified in *A. nidulans* (Shaw and Momany, 2002) and *P. digitatum* (Harries et al., 2015), suggesting an important role of MoPmt2 in regulating of the distribution of chitin content in *M. oryzae*. In addition, pathogenic test also revealed that *MoPmt2* mutants showed restricted invasive growth in host cells. Most invasive hyphae of *MoPmt2* mutant were confined to the host cells at early infection stage and the disease lesions (< 10 days) of the *MoPmt2* mutant seldom produce conidia, indicating a possible necrotic reaction and low level invasive hyphae growth of *MoPmt2* mutants in plant cells. These above results are similar to the previous findings that deletion of a α-1, 3-Mannosyltransferase encoding gene *ALG3*, which is essential for *N*-glycosylation of secreted effector like Slp1, resulted in the arrest of secondary infection hyphae and a significant reduction in virulence, and thus made us presume that the constrained growth of *MoPmt2* mutants might be ascribed to the failure of evading host innate immunity and thus attenuated virulence on host. However, in view of the facts that the *MoPmt2* mutant is defective in polarity, and some large lesions of *MoPmt2* mutant can produce conidia, we couldn't exclude that the hindered hyphae growth might also partly affect the proliferation of invasive hyphae and lesion development by the *MoPmt2* mutants.

In *M. oryzae*, conidiogenesis is a complex process that involves a series of morphological events (Liu et al., 2010). In field condition, the severity of the disease epidemic lies on the quantity of conidia produced in the rice blast lesion, suggesting an important role of conidiation in the disease cycle. Previous studies in filamentous fungus showed that disruption of *Pmt2* genes in *A. nidulans, A. fumigatus* and *P. digitatum* equally led to a significant reduction of conidiation (Goto et al., 2009; Fang et al., 2010; Harries et al., 2015), whereas in the *B. cinerea*, deletion of *Pmt2* gene resulted in a complete loss of sporulation (Gonzalez et al., 2013), indicating a potential role of *Pmt2* gene in conidial development. In this study, we observed an increased expression level of *MoPmt2* in conidia, which is similar to the transcriptional pattern of *Moap1* in *M. oryzae*, demonstrating a potential role in asexual spore development (Guo et al., 2011). This hypothesis was confirmed by the analysis of *MoPmt2* mutants, with phenotypes of significant reduction of conidiation, abnormal conidia size and delayed conidial germination, which is consistent with the identification in other fungi (Gonzalez et al., 2013; Wang et al., 2014; Harries et al., 2015), suggesting a conserved role of MoPmt2 protein in asexual spore development in *M. oryzae*. In particular, it is pointed out that conidial morphology of large conidia from the *MoPmt2* mutants, was similar to those observed in *Moap1* mutants, demonstrating that the MoPmt2 protein might function downstream of the MoAp1 and is responsible for conidial development (Guo et al., 2011).

The initial stages of infection by *M. oryzae* usually require the immediate and persistent adhesion of a conidium to the rice leaf by means of the spore tip mucilage released from the spore apex (Jelitto et al., 1994). Cell wall glycoproteins have been identified as fungal adhesives and have been implicated in host cell adhesion in many organisms (Gaur and Klotz, 1997; Frieman et al., 2002; Harries et al., 2015). *O*-mannosylation, a type of protein *O*-glycosylation with the capacity of addition of mannose residues to target proteins, have been described for fungal development, including in cell wall integrity, cell morphology and cell adhesion (Fernández-Álvarez et al., 2009; Kriangkripipat and Momany, 2009; Lommel and Strahl, 2009; Harries et al., 2015). In this study, we identified MoPmt2 as a homolog of the yeast Pmt2 protein, an *O*-mannosyltransferase from PMT2 subfamily, in *M. oryzae*. Deletion of *MoPmt2* resulted in reduced ability of conidial adhesion to hydrophobic surfaces, and thus attenuated virulence on rice plants, which agreed with the findings in *B. cinerea, A. fumigatus* (Kriangkripipat and Momany, 2009; Gonzalez et al., 2013), indicating a conserved role of MoPmt2 in fungal adhesion during plant infection. Our subsequent FITC-ConA staining revealed that STM secretion was not detected in the *MoPmt2* mutants, compared to wild-type Guy11, making us further confirm that *MoPmt2* might be involved in synthesis and secretion of STM in *M. oryzae*. However, this seems paradoxical to the result that secretion of STM from mature appressoria of *MoPmt2* mutant was comparable to that of Guy11. In *M. oryzae*, previous studies showed that STM is stored at the conidial apex of dormant conidia, and its release is an early event before germ tube emergence (Hamer et al., 1988). Therefore, we speculated that *MoPmt2* deletion might be lead to defects on storage and secretion of STM in *M. oryzae*, and our subsequent staining of mature appressoria with FITC-ConA might be not reflected the true STM, but newly synthesized glycosylated-proteins that were secreted outside of the appressorium cell wall of *MoPmt2* mutants and wild-type Guy11.

In *M. oryzae*, the fungal cell wall provides mechanical protection against attack from the host during host–pathogen interactions, and an intact cell wall is the guarantee for full virulence on rice plants (Jeon et al., 2008; Guo et al., 2015). In this study, we identified that defects on appressorium-mediated penetration was one of the main reasons for attenuated virulence of MoPmt2 mutants (Figure 7B). Our subsequent assay revealed that the MoPmt2 mutants showed increased sensitivity to CFW and CR (Figure 8), and more protoplast was released in the mutants as compared to Guy11, suggesting a defective CWI in the MoPmt2 mutants (Figure 8). This deduction was supported by the analysis of turgor pressure, with more collapsed appressorium being observed in the MoPmt2 mutants at different glycerol solutions. Together with the altered distribution of chitin content in the cell wall of both mycelia and appressorium, we concluded that the defective CWI in the mutants might be responsible for their inability to accumulate sufficient turgor pressure in appressorium, thus leading to failed penetration of host cells and attenuated pathogenicity on rice plants. Our results is consistent with the biological roles of *Pmt2* in phytopathogen like *B. cinerea*, B. bassiana and *P. digitatum*, which is critical for the stability of cell wall integrity (Gonzalez et al., 2013; Wang et al., 2014; Harries et al., 2015).

In phytopathogens, secreted peroxidases are regarded to help pathogens to detoxify host-derived ROS during plant-microbe interactions (Chi et al., 2009; Guo et al., 2010, 2011). We identified an attenuation of secreted peroxidase activity in the *MoPmt2* mutant by comparing CR discoloration and assaying the peroxidase activity in culture filtrates. Meanwhile, the secreted laccases were also reduced in enzyme activity in the culture filtrates of *MoPmt2* mutant. In *M. oryzae*, a previous study revealed that hundreds of putatively secretory proteins possessed Ser/Thr-rich regions and could be potentially *O*-glycosylated in protein posttranslational modification (Gonzalez et al., 2012), thus, combined with the observed phenotypes, we presumed that MoPmt2 may be required for the *O*-glycosylation of those secreted peroxidases and laccases, and its deletion may result in proteins modification defects, which thus reduced the enzymatic activity of the *MoPmt2* mutants.

In summary, our result reveals that the MoPmt2 play a critical role during the development of *M. oryzae*. The deletion of *MoPmt2* could result in defects on conidiation, fungal adhesion, conidia germination, CWI and invasive hyphae growth, and thus attenuated the pathogenicity of *M. oryzae* on rice plants.

## Author contributions

Conceived and designed the experiments: MG, ZG. Performed the experiments: MG, LT, XN. Analyzed the data: MG, XZ, XN, YP. Wrote the paper: MG.

### Conflict of interest statement

The authors declare that the research was conducted in the absence of any commercial or financial relationships that could be construed as a potential conflict of interest.

## Supplementary material

Figure S1 **The dendrogram of PMT proteins from different organisms**. A phylogenetic tree of MoPmts homologs was created by the distance based minimum evolution method, based on 1000 bootstraps. *M. oryzae* sequences characterized in this study are highlighted in dotted box. Pmt proteins from different fungal organisms were obtained from NCBI database (http://www.ncbi.nlm.nih.gov/) and the accession numbers of all the sequences used in this analysis are as followings: *M. oryzae* (XP_003720753, ELQ38305, XP_003713520), *S. cerevisiae* (NP_010188, NP_009379, NP_014966, NP_012677, NP_010190, NP_011715), *C. albicans* (XP_716993, XP_719907, XP_714280, XP_719311, XP_717283), *S. pombe* (NP_593237, NP_594135, NP_596807), *U. maydis* (XP_762320, XP_761621, XP_761580), *Drosophila melanogaster* (NP_524025, NP_569858), *Homo sapiens* (NP_009102.3, NP_037514.2), *C. neoformans* (XP_570521, XP_567365, XP_570292), *B. cinerea* (XP_001548518, XP_001558317, XP_001558914), *A. nidulans* (XP_662365, XP_662709, XP_659063), *B. bassiana* (EJP63368, EJP63582, EJP70423), *Metarhizium anisopliae* (EFY94173, EFZ02257, EFZ00337), *Aspergillus niger* (XP_001394947, XP_001392110, XP_001398147), *A. fumigates* (EAL92923, XP_754961, XP_747257), *Penicillium mameffei* (EEA19578, EEA22196), *Neurospora crassa* (XM_960450, XM_951177, XM_958833), *P. digitatum* (KC757712, KC757713, KC757714), and *Penicillium crysogenum* (AM920435, AM920427, AM920436).

Figure S2 **The relative expression, targeted gene replacement and complementation of *MoPmt2*. (A)** *MoPmt2* expression at different developmental stage. Significant differences are presented in the figure (*P* < 0.01), and the error bar represents the standard deviation. **(B)** Targeted gene replacement strategies. The DNA fragment deleted from the *MoPmt2* region was used as the probe to validate the deletion of *MoPmt2* by Southern blot (Scale bar = 1 kb). **(C)** Validation of the transformants by PCR amplification. Primer pairs (p1 to p4) showed in this figure were used to validate the transformants by PCR amplification. **(D,E)**. Southern blot analysis. To confirm the copy number of *MoPmt2* gene in Guy11 and the deletion of *MoPmt2* gene in the mutants, both the genomic DNA of Guy11 and the mutants, which were digested with *Sal*I, respectively, were hybridized with probe of *MoPmt2* left flank. To validate the integration of a single copy of *HPH* gene in the mutants, genomic DNA of Guy11 and *MoPmt2* mutants were digested with *Sal*I, and hybridized with *HPH* probe. **(F)** Semiquantitative RT-PCR. RNA samples from Guy11, *MoPmt2* mutants and *MoPmt2c* were reversely transcripted and used to confirm the deletion and reintroduction of the *MoPmt2* gene by PCR amplification.

Figure S3 **Mycelial growth of the *MoPmt2* mutants on different media**. The wild-type strain Guy11, *MoPmt2* mutants and complemented strain *MoPmt2c* was inoculated on CM, MM, OM, and RDC, and cultured at 28°C for 5 days.

Figure S4 **Conidia bipolar germination in *MoPmt2* mutants. (A)** Polarity growth defects of *MoPmt2* mutants. CFW staining of mycelia show polarity growth defects and swollen of mycelia of *MoPmt2* mutants. **(B)** Conidial suspension (1 × 10^5^ ml^−1^) of Guy11, *MoPmt2-6* mutant and *MoPmt2c*, harvested from 14-day-old cultures, were inoculated on the hydrophobic surface of the coverslips for 24 h, and then observed under light microscope. **(C)** Statistical analysis of conidia with bipolar germination. The percentage of conidia with unipolar and bipolar germination was calculated and statistically analyzed, respectively.

Figure S5 **Conidial morphology of *MoPmt2* mutants on conidiophores**. The development of conidia on conidiophores was examined by light microscope using strains grown on RDC medium for 7 days. Scale bar = 30 μm.

Figure S6 **The staining of appressorium with FITC-ConA or CFW. (A)** Mucilage secreted from the appressorium of Guy11 and *MoPmt2* mutants were stained with FITC-ConA. The fluorescence signal from mature appressoria were visualized by a Nikon inverted T*i*-S epifluorescence microscope. **(B)** The CFW staining of appressorium of Guy11, *MoPmt2* mutant and *MoPmt2c*. The fluorescence signal from mature appressoria were captured by a Nikon inverted T*i*-S epifluorescence microscope.

Figure S7 **Decreased extracellular laccase and peroxidase activities in the *MoPmt2* mutants**. Guy11, *MoPmt2* mutants and *MoPmt2c* were inoculated in CM liquid medium and the peroxidase activity **(A)** and laccase activity **(B)** were measured in the filtrate cultures through ABTS oxidization test with or without H_2_O_2_. Error bars represent the standard deviations and asterisks represent significant differences among the strains tested (*p* < 0.01).

Table S1 **Bioinformatics of putative *M. oryzae* PMT proteins**.

Table S2 **Primers used in this paper**.
