# Supplementary figures and images for: The Pmt2p-Mediated Protein O-Mannosylation Is Required for Morphogenesis, Adhesive Properties, Cell Wall Integrity and Full Virulence of Magnaporthe oryzae
Source: Front Microbiol. 2016 May 2;7:630. doi: 10.3389/fmicb.2016.00630 (PMC4852298; doi:10.3389/fmicb.2016.00630)

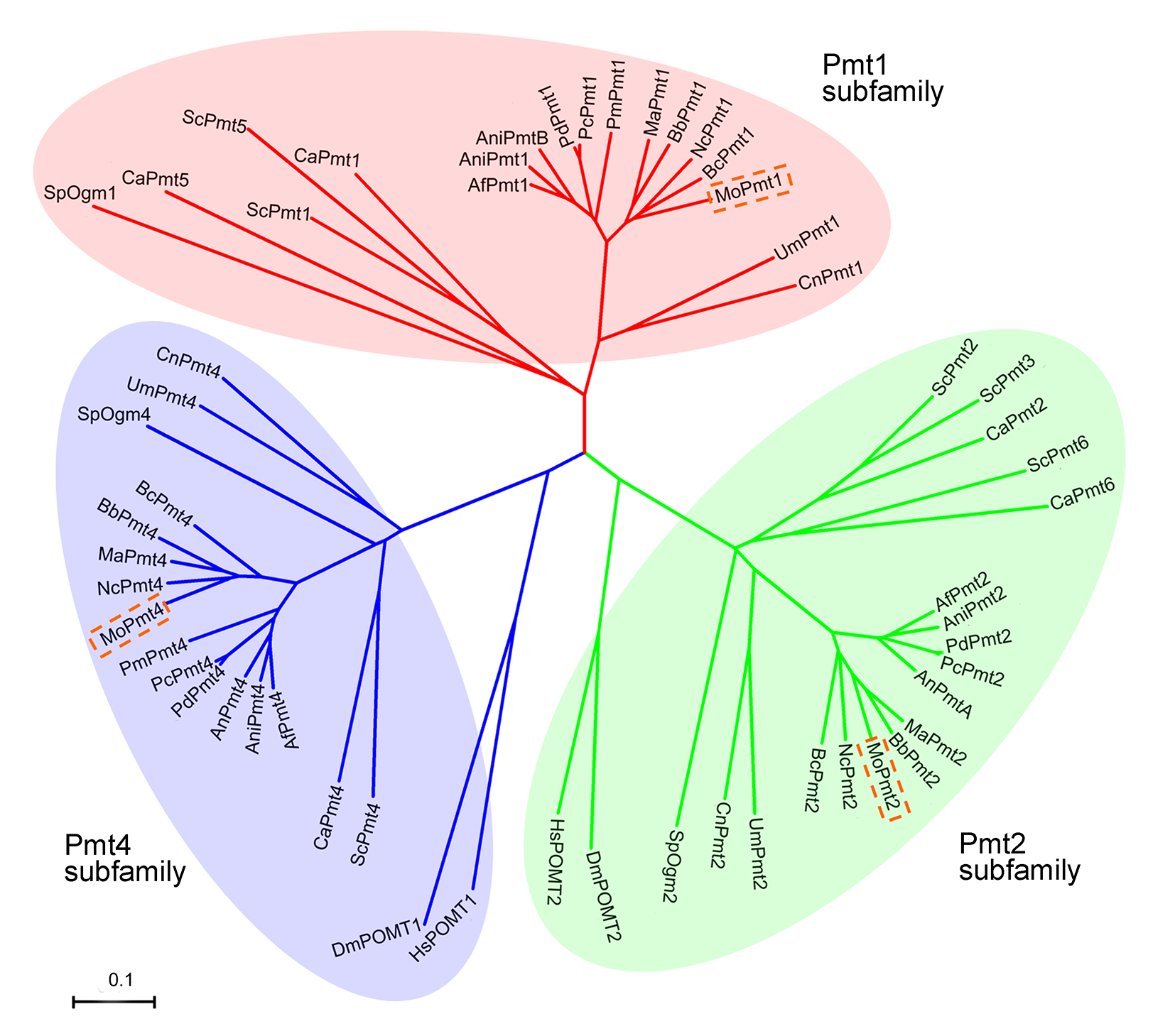

Supplement: Figure S1 — The dendrogram of PMT proteins from different organisms. A phylogenetic tree of MoPmts homologs was created by the distance based minimum evolution method, based on 1000 bootstraps. M. oryzae sequences characterized in this study are highlighted in dotted box. Pmt proteins from different fungal organisms were obtained from NCBI database (http://www.ncbi.nlm.nih.gov/) and the accession numbers of all the sequences used in this analysis are as followings: M. oryzae (XP_003720753, ELQ38305, XP_003713520), S. cerevisiae (NP_010188, NP_009379, NP_014966, NP_012677, NP_010190, NP_011715), C. albicans (XP_716993, XP_719907, XP_714280, XP_719311, XP_717283), S. pombe (NP_593237, NP_594135, NP_596807), U. maydis (XP_762320, XP_761621, XP_761580), Drosophila melanogaster (NP_524025, NP_569858), Homo sapiens (NP_009102.3, NP_037514.2), C. neoformans (XP_570521, XP_567365, XP_570292), B. cinerea (XP_001548518, XP_001558317, XP_001558914), A. nidulans (XP_662365, XP_662709, XP_659063), B. bassiana (EJP63368, EJP63582, EJP70423), Metarhizium anisopliae (EFY94173, EFZ02257, EFZ00337), Aspergillus niger (XP_001394947, XP_001392110, XP_001398147), A. fumigates (EAL92923, XP_754961, XP_747257), Penicillium mameffei (EEA19578, EEA22196), Neurospora crassa (XM_960450, XM_951177, XM_958833), P. digitatum (KC757712, KC757713, KC757714), and Penicillium crysogenum (AM920435, AM920427, AM920436). [file Image1.TIF]

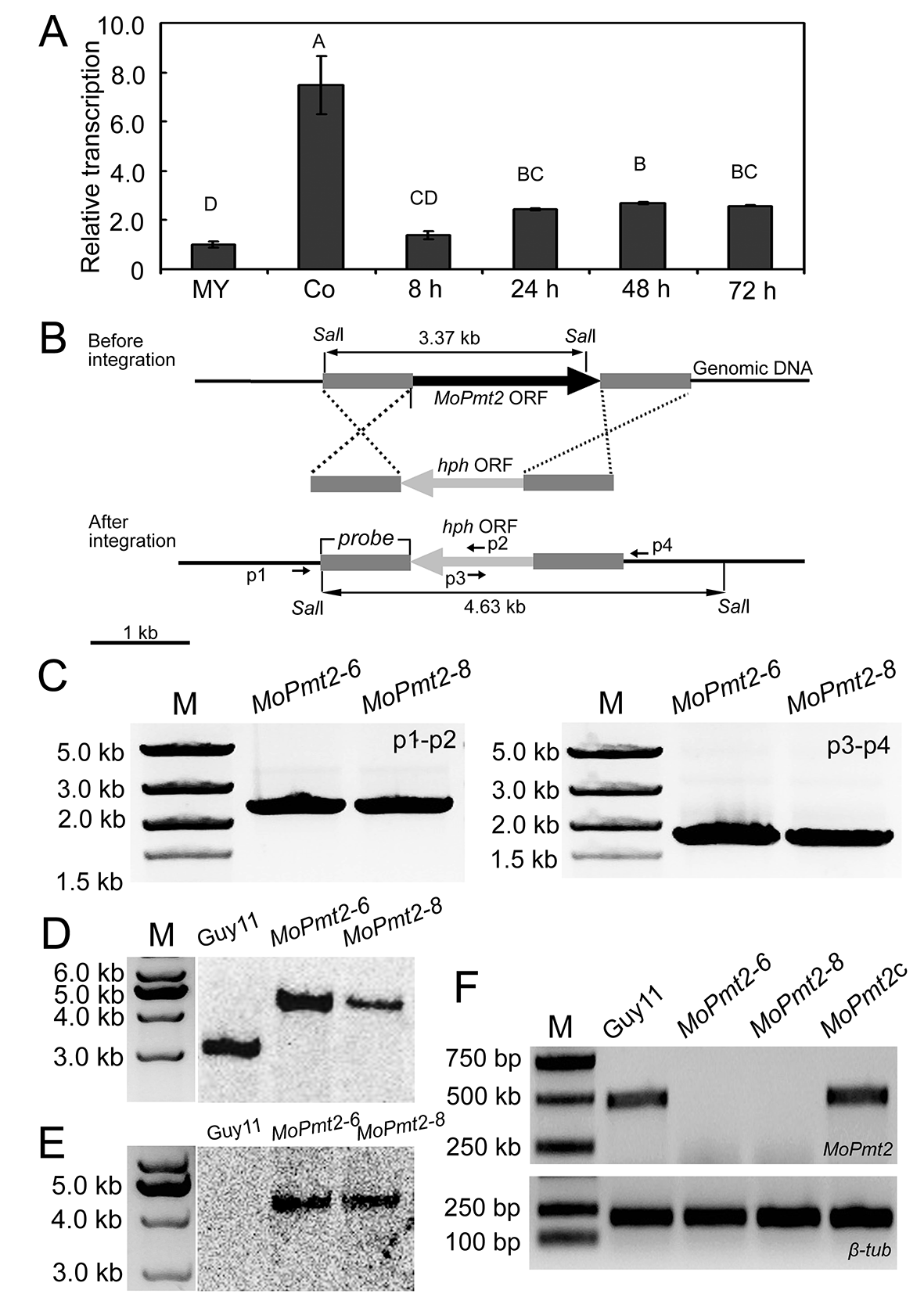

Supplement: Figure S2 — The relative expression, targeted gene replacement and complementation of MoPmt2. (A) MoPmt2 expression at different developmental stage. Significant differences are presented in the figure (P < 0.01), and the error bar represents the standard deviation. (B) Targeted gene replacement strategies. The DNA fragment deleted from the MoPmt2 region was used as the probe to validate the deletion of MoPmt2 by Southern blot (Scale bar = 1 kb). (C) Validation of the transformants by PCR amplification. Primer pairs (p1 to p4) showed in this figure were used to validate the transformants by PCR amplification. (D,E). Southern blot analysis. To confirm the copy number of MoPmt2 gene in Guy11 and the deletion of MoPmt2 gene in the mutants, both the genomic DNA of Guy11 and the mutants, which were digested with SalI, respectively, were hybridized with probe of MoPmt2 left flank. To validate the integration of a single copy of HPH gene in the mutants, genomic DNA of Guy11 and MoPmt2 mutants were digested with SalI, and hybridized with HPH probe. (F) Semiquantitative RT-PCR. RNA samples from Guy11, MoPmt2 mutants and MoPmt2c were reversely transcripted and used to confirm the deletion and reintroduction of the MoPmt2 gene by PCR amplification. [file Image2.TIF]

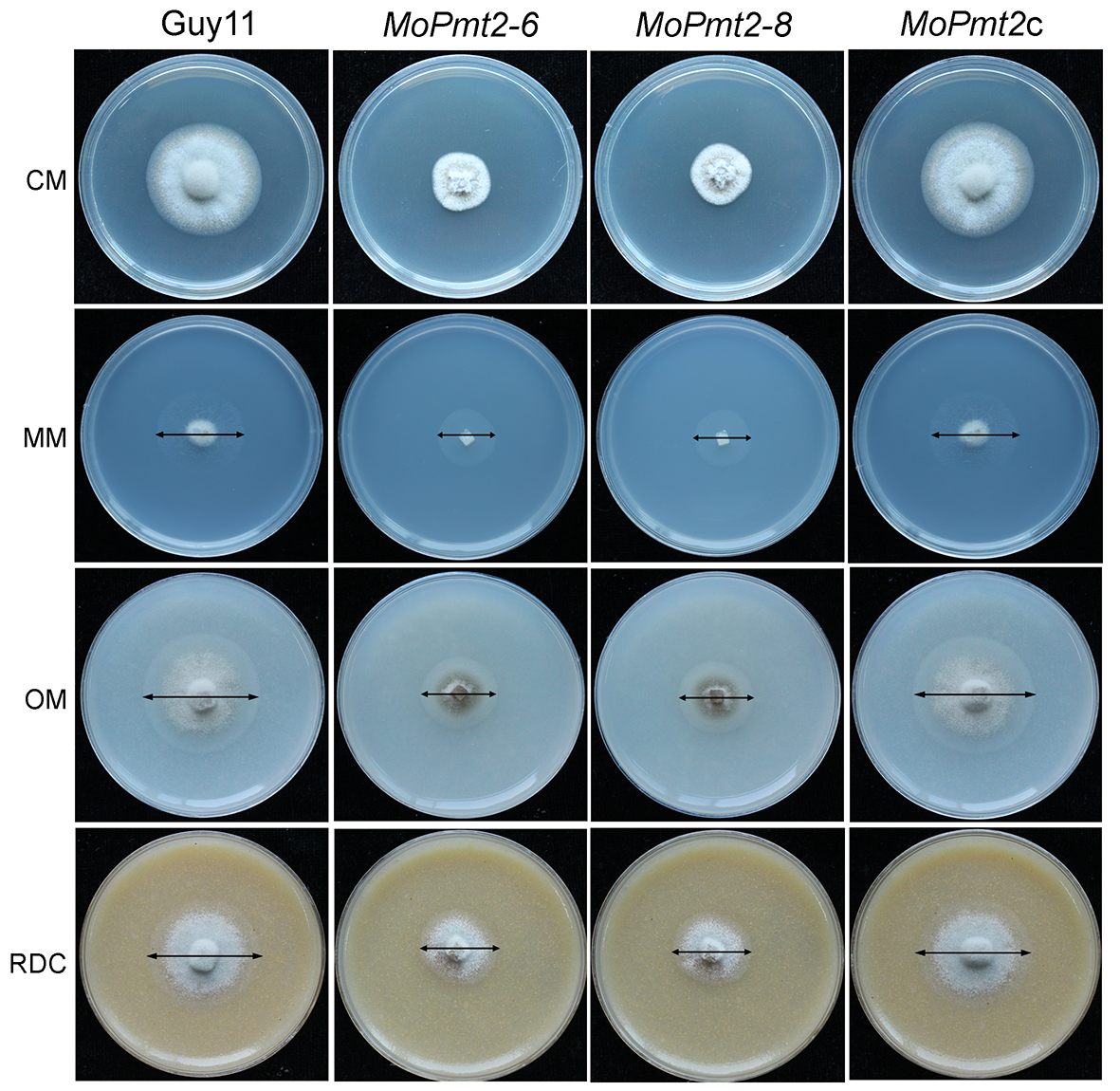

Supplement: Figure S3 — Mycelial growth of the MoPmt2 mutants on different media. The wild-type strain Guy11, MoPmt2 mutants and complemented strain MoPmt2c was inoculated on CM, MM, OM, and RDC, and cultured at 28°C for 5 days. [file Image3.TIF]

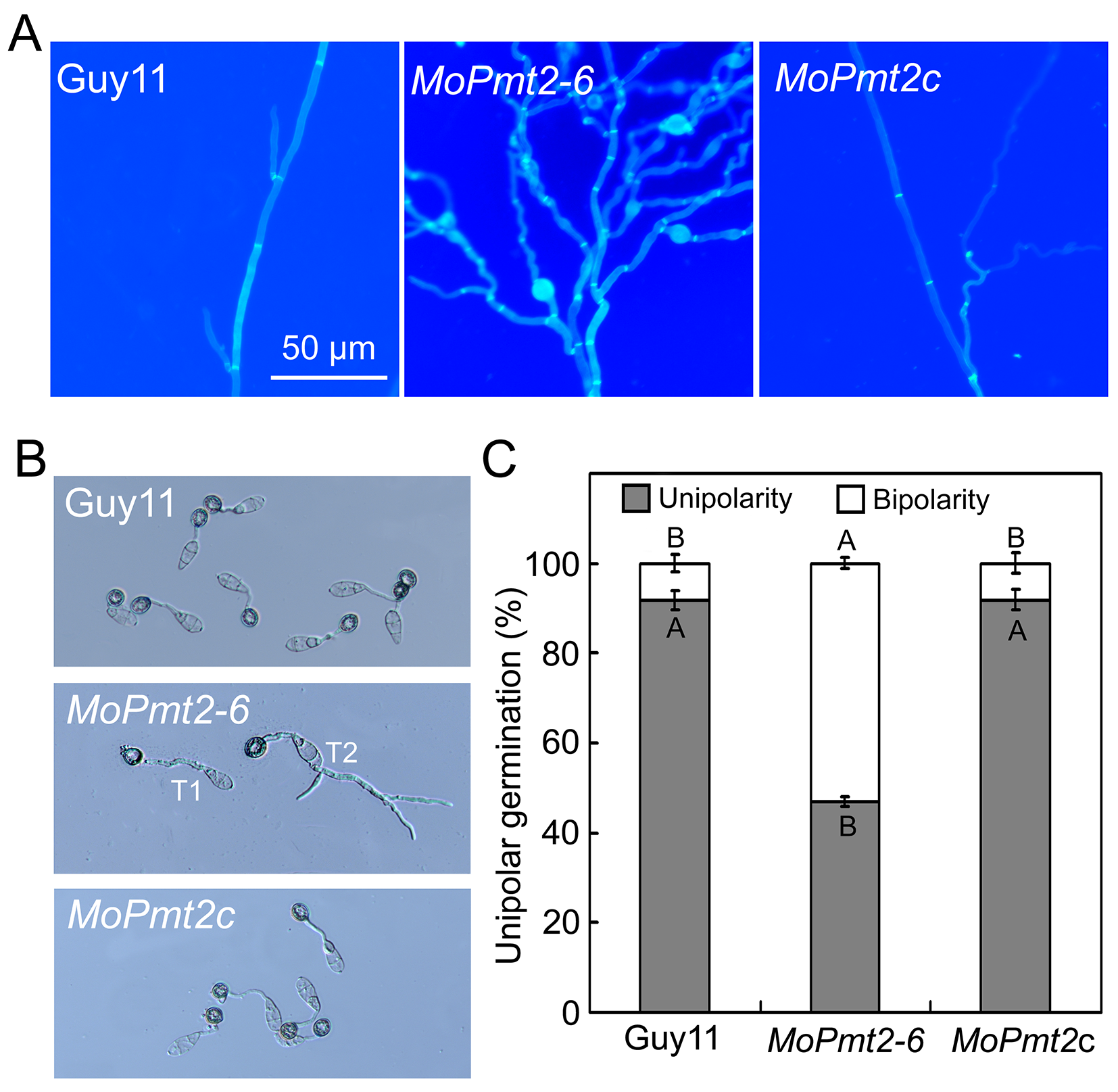

Supplement: Figure S4 — Conidia bipolar germination in MoPmt2 mutants. (A) Polarity growth defects of MoPmt2 mutants. CFW staining of mycelia show polarity growth defects and swollen of mycelia of MoPmt2 mutants. (B) Conidial suspension (1 × 105 ml−1) of Guy11, MoPmt2-6 mutant and MoPmt2c, harvested from 14-day-old cultures, were inoculated on the hydrophobic surface of the coverslips for 24 h, and then observed under light microscope. (C) Statistical analysis of conidia with bipolar germination. The percentage of conidia with unipolar and bipolar germination was calculated and statistically analyzed, respectively. [file Image4.TIF]

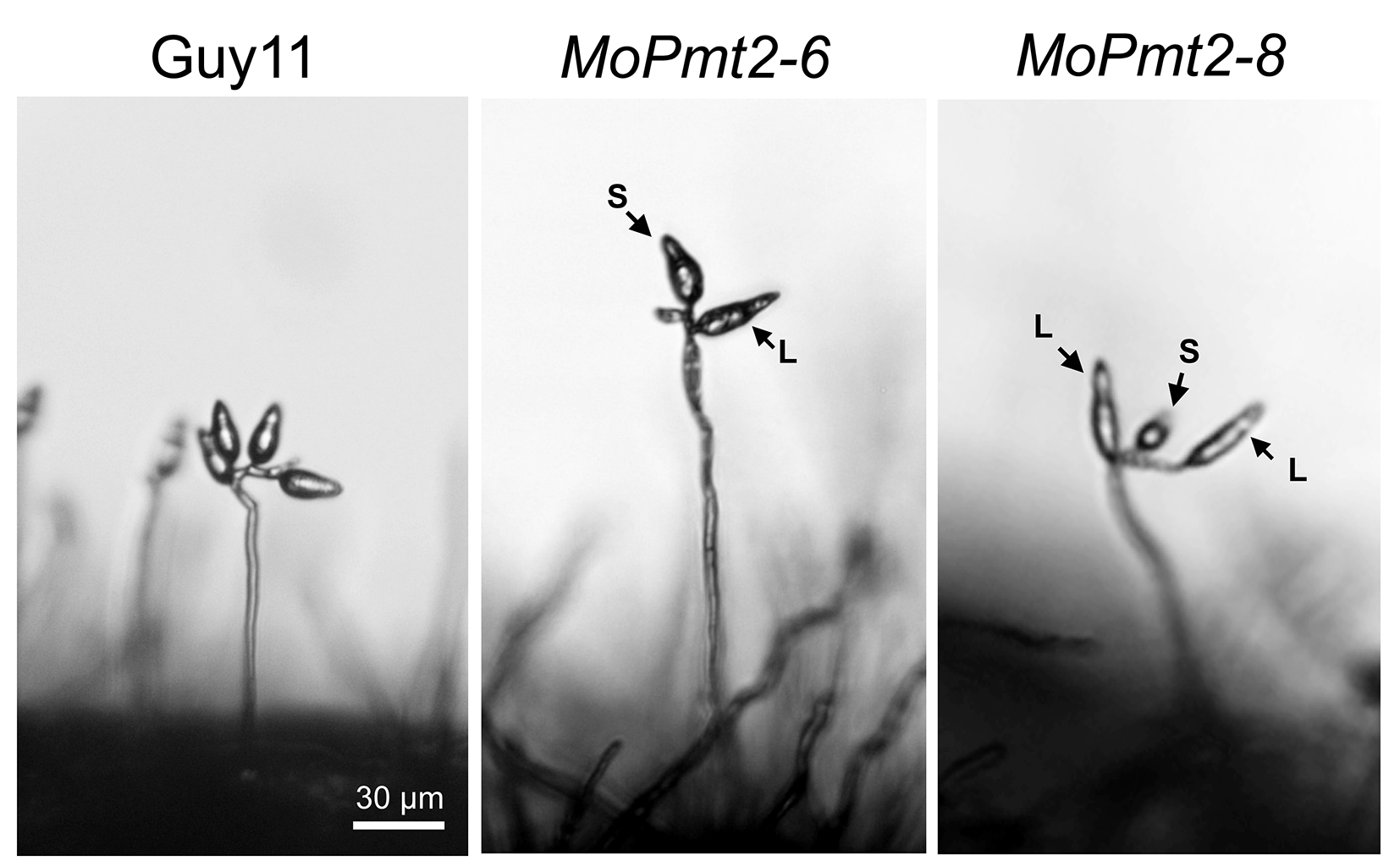

Supplement: Figure S5 — Conidial morphology of MoPmt2 mutants on conidiophores. The development of conidia on conidiophores was examined by light microscope using strains grown on RDC medium for 7 days. Scale bar = 30 μm. [file Image5.TIF]

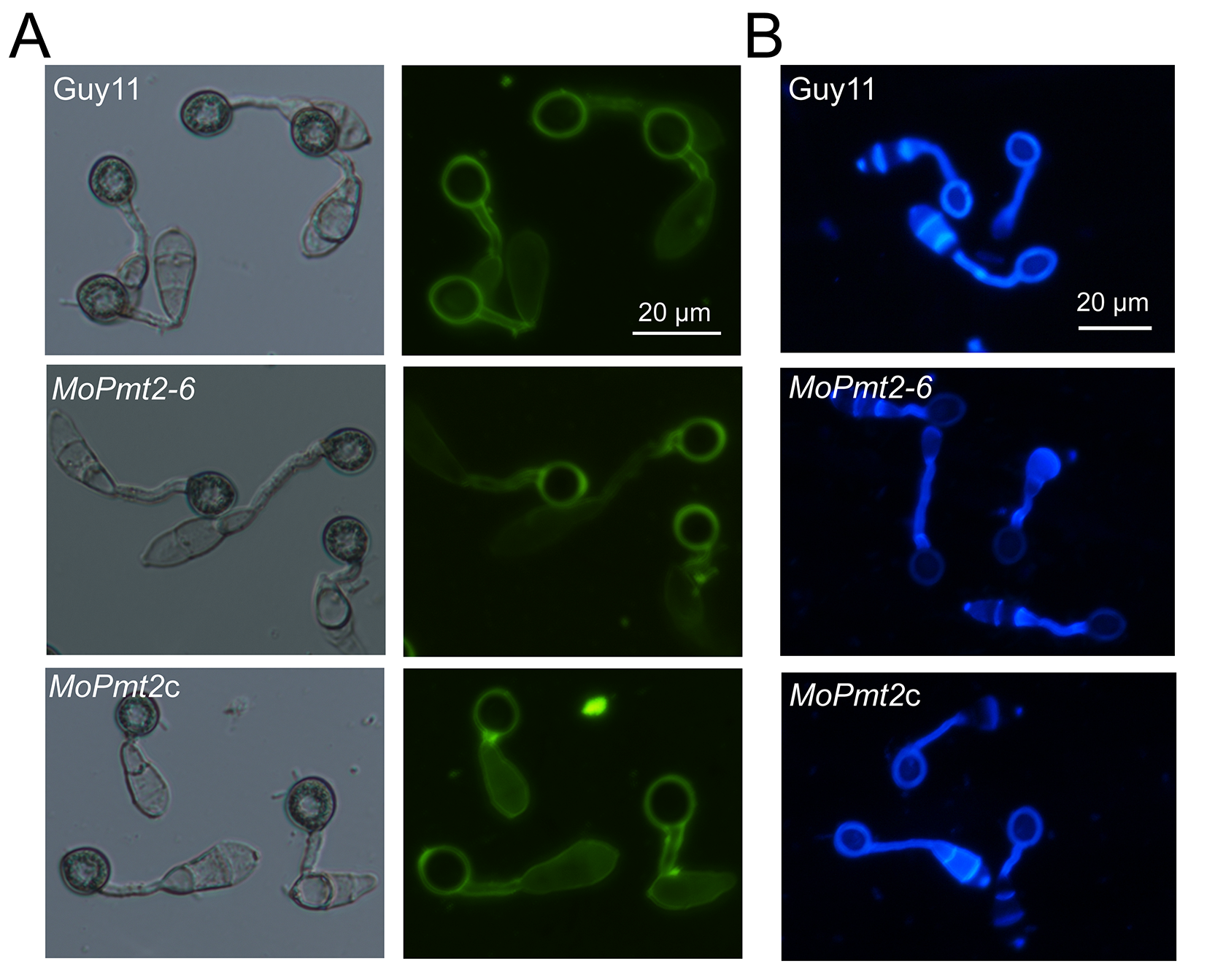

Supplement: Figure S6 — The staining of appressorium with FITC-ConA or CFW. (A) Mucilage secreted from the appressorium of Guy11 and MoPmt2 mutants were stained with FITC-ConA. The fluorescence signal from mature appressoria were visualized by a Nikon inverted Ti-S epifluorescence microscope. (B) The CFW staining of appressorium of Guy11, MoPmt2 mutant and MoPmt2c. The fluorescence signal from mature appressoria were captured by a Nikon inverted Ti-S epifluorescence microscope. [file Image6.TIF]

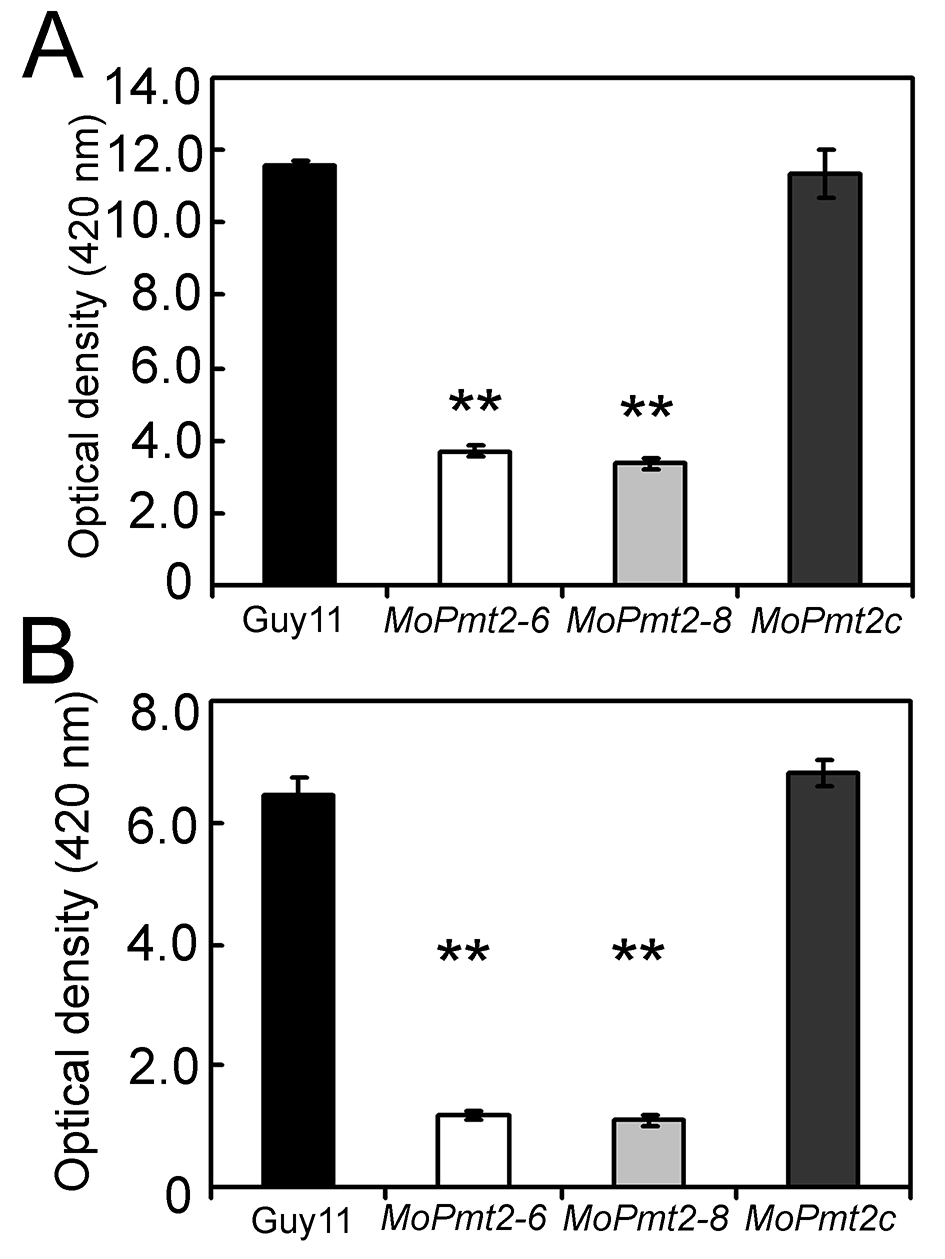

Supplement: Figure S7 — Decreased extracellular laccase and peroxidase activities in the MoPmt2 mutants. Guy11, MoPmt2 mutants and MoPmt2c were inoculated in CM liquid medium and the peroxidase activity (A) and laccase activity (B) were measured in the filtrate cultures through ABTS oxidization test with or without H2O2. Error bars represent the standard deviations and asterisks represent significant differences among the strains tested (p < 0.01). [file Image7.TIF]
