# Supplementary material for: The Pmt2p-Mediated Protein O-Mannosylation Is Required for Morphogenesis, Adhesive Properties, Cell Wall Integrity and Full Virulence of Magnaporthe oryzae
Source: Front Microbiol. 2016 May 2;7:630. doi: 10.3389/fmicb.2016.00630 (PMC4852298; doi:10.3389/fmicb.2016.00630)
Supplement: Table S1 — Bioinformatics of putative M. oryzae PMT proteins. [file Table1.DOC]

Table S1

Bioinformatics of putative *M. oryzae* PMT proteins.

| Gene name | Length | | Domains^a^ | | Identity^b^ | Reference^c^ |
| --- | --- | --- | --- | --- | --- | --- |
| PMT1 | *S. cerevisiae*  817 aa | *M. oryzae*  997 aa | *S. cerevisiae*  PMT domain (54 to 297; 4.4e-91)  MIR domain (343 to 517; 3.6e-50)  TMM region (533 to 717;1.4e-58) | *M. oryzae*  PMT domain (66 to 317; 1.1e-77)  MIR domain (362 to 551; 1.7e-48)  TMM region (564 to 762; 5.2e-60) | 39% | Gentzsch et al.,1996 EMBO J. 15: 5752–5759 |
| PMT2 | *S. cerevisiae*  759 aa | *M. oryzae*  736 aa | *S. cerevisiae*  PMT domain (69 to 313; 2.3e-99)  MIR domain (358 to 525; 3.4e-24)  TMM region (548 to 751; 1e-60) | *M. oryzae*  PMT domain (59 to 303; 1.7e-81)  MIR domain (350 to 511; 1.8e-37)  TMM region (535to 732; 2.5e-63) | 47% | Gentzsch et al.,1996 EMBO J. 15: 5752–5759 |
| PMT4 | *S. cerevisiae*  762 aa | *M. oryzae*  774 aa | *S. cerevisiae*  PMT domain (57 to 305; 2.6e-88)  MIR domain (341 to 516; 1.9e-21)  TMM region (541to 756; 4.8e-56) | *M. oryzae*  PMT domain (57 to 307; 4.8e-84)  MIR domain (351 to 517; 9e-21)  TMM region (542 to 768; 1.4e-58) | 45% | Gentzsch et al.,1996 EMBO J. 15: 5752–5759 |

a Determined in PFAM (http://pfam.xfam.org/search/sequence) with error probability in brackets.

b Determined in EMBOSS Needle (http://www.ebi.ac.uk/Tools/psa/emboss_needle/).

c Reference for comparison.
