# Supplementary material for: The Pmt2p-Mediated Protein O-Mannosylation Is Required for Morphogenesis, Adhesive Properties, Cell Wall Integrity and Full Virulence of Magnaporthe oryzae
Source: Front Microbiol. 2016 May 2;7:630. doi: 10.3389/fmicb.2016.00630 (PMC4852298; doi:10.3389/fmicb.2016.00630)
Supplement: Table S2 — Primers used in this paper. [file Table2.DOC]

Table S2 Primers used in this paper

| Primer name | Primer sequences (5´-3´) | Remark |
| --- | --- | --- |
| Pmt2-1F | ACACTTACTTGCTCGTCGACAG | *MoPmt2* deletion vector construction |
| Pmt2-1R | GACATTCTCCATGTGTCGAACCGTTTAAACTCTGACAGATTTGGCGCAAGAG |
| Pmt2-2F | CTCTTGCGCCAAATCTGTCAGAGTTTAAACGGTTCGACACATGGAGAATGTC |
| Pmt2-2R | ATTTGACGCGTACCAGCAGTAG |
| Pmt2-S1 | GCTGGTGACCTTGATGGTTCTT | RT-PCR validation of *MoPmt2* |
| Pmt2-S2 | CAACCTCTGTTCCCTGCAAGTT |
| HPH-F | GGAGGTCAACACATCAATG | *HPH* gene amplification |
| HPH-R | CGAGGGCAAAGGAATAGAG |
| P1 | ATCGCCAGAGTATGAAGAGAGG | Validation of *MoPmt2* deletion |
| P2 | GCTTTCAGCTTCGATGTAGGAG |
| P3 | CTCCTACATCGAAGCTGAAAGC |
| P4 | GTCTTCGTCGTCGTTACACTCC |
| Actin-F | TCGACGTCCGAAAGGATCTGT | Amplification of *actin* gene in qRT-PCR |
| Actin-R | ACTCCTGCTTCGAGATCCACATC |
| Pmt2-C1 | CTACCCAAACTTCGTCGGTTTCTAGGTCACATCGCCAGAGTATGAAGAGA | *MoPmt2* complementation |
| Pmt2-C2 | CCATGCATAAATGGGTGGAGATGCGTTCACGCAAGATATCCATCACGTCC |
| Pmt2-YC1 | AAGCTTATGGCCGCGGATAAC | Yeast complementation |
| Pmt2-YC2 | CTCGAGTTAATCAGACATTCTCC |
| Pmt2-qF | CGTCACCATCAAGAACATGG | MoPmt2 qRT-PCR |
| Pmt2-qR | ACGTGGATCAGGCGTAAAAC |
| β-tubF | TCGACAGCAATGGAGTTTAC | Amplification of β-*tubulin* gene in RT-PCR |
| β-tubR | AGCACCAGACTGACCGAAGAC |
| Pmt2-P1 | ACACTTACTTGCTCGTCGACAG | Southern blot probe amplification |
| Pmt2-P2 | TCTGACAGATTTGGCGCAAGAG |
